# Mesenchymal Stem Cell-Derived Exosomes as Drug Delivery Vehicles in Disease Therapy

**DOI:** 10.3390/ijms25147715

**Published:** 2024-07-14

**Authors:** Wenzhe Zhao, Kaixuan Li, Liangbo Li, Ruichen Wang, Yang Lei, Hui Yang, Leming Sun

**Affiliations:** 1 School of Life Sciences, Engineering Research Center of Chinese Ministry of Education for Biological Diagnosis, Treatment and Protection Technology and Equipment in Special Environment, Northwestern Polytechnical University, Xi’an 710072, China; 19959002885@mail.nwpu.edu.cn (W.Z.); 18732070428@mail.nwpu.edu.cn (K.L.); liliangbo@mail.nwpu.edu.cn (L.L.); wangruichen@mail.nwpu.edu.cn (R.W.); leiyang2020@mail.nwpu.edu.cn (Y.L.); 2 Dongguan Sanhang Innovation Institute, Dongguan 523808, China

**Keywords:** mesenchymal stem cell, exosome, drug delivery, nanocarriers, disease therapy

## Abstract

Exosomes are small vesicles containing proteins, nucleic acids, and biological lipids, which are responsible for intercellular communication. Studies have shown that exosomes can be utilized as effective drug delivery vehicles to accurately deliver therapeutic substances to target tissues, enhancing therapeutic effects and reducing side effects. Mesenchymal stem cells (MSCs) are a class of stem cells widely used for tissue engineering, regenerative medicine, and immunotherapy. Exosomes derived from MSCs have special immunomodulatory functions, low immunogenicity, the ability to penetrate tumor tissues, and high yield, which are expected to be engineered into efficient drug delivery systems. Despite the promising promise of MSC-derived exosomes, exploring their optimal preparation methods, drug-loading modalities, and therapeutic potential remains challenging. Therefore, this article reviews the related characteristics, preparation methods, application, and potential risks of MSC-derived exosomes as drug delivery systems in order to find potential therapeutic breakthroughs.

## 1. Introduction

Exosomes are a class of extracellular vesicles (EVs) secreted by cells between 40 and 100 nm in diameter [1,2]. For a long time, exosomes were considered as a way for cells to discard unwanted proteins, biomolecules, and other waste products, so they were also called “garbage bags” [3]. Johnstone first defined these EVs as “exosomes” in 1987 [4]. With the continuous deepening of research, exosomes were found to be capable of intracellular communication by transferring mRNA and *microRNA* (miRNA) in 2007 [5], which attracted wide attention from scientists. Soon after that, regulating the transport of vesicles inside cells won the 2013 Nobel Prize in Physiology or Medicine [6], which represented a whole new level of exosome research.

Exosomes are rich in sources and can be extracted from normal cells, cancer cells, immune cells [7], etc. Among them, MSCs are one of the most widely used cells because of their ability to self-renew and multidirectional differentiation [8]. The source of MSCs is abundant, which can be isolated from various tissues such as bone marrow and fat, and has good proliferation ability in vitro [9]. Although MSCs were widely used for clinical testing because of their potential regenerative power, several studies have shown that implanted MSCs rarely differentiate and proliferate into appropriate cell types [10,11,12]. In 1996, Haynesworth et al. [13] reported that MSCs can synthesize and secrete growth factors, chemokines, and cytokines that have significant effects on their surrounding cells. This was the first report on the paracrine effect of MSCs. Many subsequent studies have provided strong evidence for the paracrine hypothesis of MSCs [14,15,16,17] and proved that the primary regeneration mode, which involves the proliferation of preexisting cells of MSC transplantation is a paracrine effect rather than the differentiation effect [18,19]. MSCs can secrete a variety of EVs, including exosomes, microvesicles, and apoptotic bodies [20]. Among them, exosomes are the best characterized and have more definitive biophysical and biochemical parameters [21]. Lai et al. [22] first discovered MSC-derived exosomes when separating an MSC culture medium. 

There are many ways to extract the exosomes derived from MSCs. Currently, the commonly used methods include ultracentrifugation, ultrafiltration, size exclusion chromatography, precipitation with hydrophilic polymers, equidensity gradient centrifugation, immunoaffinity capture, etc. [23,24,25]. MSC-derived exosomes have special immunomodulatory functions, high production, and low cost, and have been demonstrated to have similar biological functions to MSCs [26]. In the meantime, they have the function of penetrating tumor tissues, which is very beneficial for the delivery of therapeutic agents [26]. For all these reasons, MSC-derived exosomes are easily constructed into cell lines with a stable expression, thus becoming an ideal tool for specific cell-targeted drug delivery [27,28]. In fact, many studies have focused on using exosomes derived from MSCs for drug delivery to treat or detect diseases. For example, Chen et al. [29] found that in a rat model of acute ischemic stroke, mini pig adipose-derived MSCs and their derived exosomes could reduce the cerebral infarction area in rats. In the study of Liang et al. [30], LncRNA KLF3-AS1 in exosomes secreted from human-MSCs (hMSCs) by acting as a ceRNA to sponge miR-138-5p can regulate Sirt1 so as to inhibit cell pyroptosis and attenuate myocardial infarction (MI) progression.

**Figure 1:**
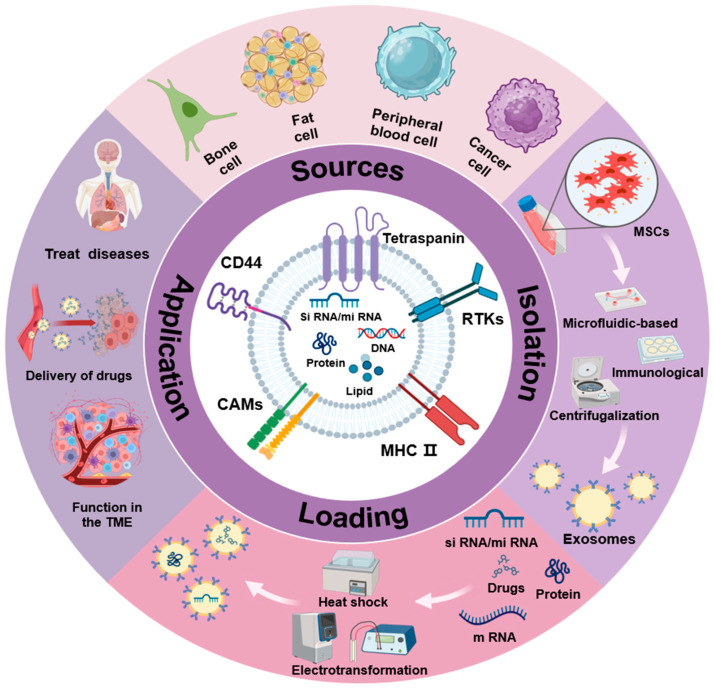
MSC-derived exosomes as drug delivery vehicles in disease.

In this review, we will focus on the related characteristics, preparation methods, research status, and potential risks of MSC-derived exosomes as drug delivery carriers and elaborate on the application of MSC-derived exosomes as drug delivery vehicles in disease monitoring and treatment (Figure 1), while hoping to provide enlightenment for the development of disease treatment strategies in the future.

## 2. MSC-Derived Exosomes

### 2.1. Biogenesis of EVs

EV generation is a complex cell biological process involving several vital steps such as synthesis, modification, endosome formation, transport, and release. Typically, MSC-derived exosomes are the result of multiple endocytosis and membrane fusion [31]. When the signaling molecules in the cell are triggered to the cell, the plasma membrane will puff inward to form an endocytic vesicle. At the same time, activation of relevant signaling pathways induces the endoplasmic reticulum and Golgi apparatus to synthesize proteins, nucleic acids, signaling molecules, and other goods carried by these vesicles. The vesicles formed by endocytosis of the plasma membrane merge with each other and enter the related organelles for modification to form intraluminal vesicles (ILVs). ILVs form a multivesicular body (MVB) after completing cargo sorting in the endoplasmic reticulum [32], containing various proteins, nucleic acids, etc. Two fates await MVBs: either it fuses with the lysosome to degrade the cargo or fuses with the plasma membrane to successfully secrete it outside the cell as an exosome [33].

Cargo sorting is an essential step in the formation of MVBs, which is regulated by many pathways and molecules. The endosomal sorting complexes required for transport (ESCRT) is an important group of proteins involved in this process, relying mainly on five core complexes: ESCRT-0, ESCRT-I, ESCRT-II, ESCRT-III, and Vps4-Vtal complexes [34]. MVB formation can be divided into ESCRT-dependent and ESCRT-independent pathways according to ESCRT [35]. In addition to differences in pathways, the sorting mechanism is also related to the type of cargo. For example, the classical signal of the protein joining the endosome pathway is monoubiquitination [33]. This means that once the protein is labeled with ubiquitin, a special domain called ubiquitin-binding domain (UBD) contained in the subunits of the ESCRT complex will recognize and bind the ubiquitin-labeled protein, sorting it into the appropriate MVB. However, not all proteins are sorted through the ESCRT-dependent pathway, and some proteins can also be directed to lysosomes for degradation through lipids or direct interactions with other proteins [36].

The generation of MSC-derived exosomes is complex and involves a variety of biomolecules regulated by different pathways [31]. We enumerated a subset of the biomolecules that regulate their generation (Table 1). A deeper understanding of the molecular mechanisms of this EV biogenesis will help to understand the role of exosomes more fully in physiological and pathological processes, providing possible options for intervention therapy.

### 2.2. Structure of MSC-Derived Exosomes

MSC-derived exosomes are one class with distinct characteristics among the EVs secreted by MSCs. These EVs exhibit complex and elaborate structures that contribute to their stability, cargo protection, and cell targeting. MSC-derived exosomes typically have a diameter between 40 and 100 nm and are able to concentrate at sucrose levels between 1.1 and 1.18 g/ml [62]. The outer layer is covered by a lipid bilayer, which contains exosomal marker proteins such as MHC II, Tsg101, and CD29, and tetraspanins such as CD9 and CD81 [63]. In addition to exosomal marker proteins, MSC-derived exosomes also have CD29, CD73, CD90, CD44, and CD105, which are characteristic markers of MSCs [64].

A considerable number of studies have reported the proteomic characteristics of MSC-EVs. Lai et al. [65] confirmed through correlation analysis that MSC-derived exosomes were rich in Gil ganglioside (an endogenous receptor of the cholera toxin B chain), so MSC-derived exosomes could be distinguished from the three types of EVs. Meanwhile, functional analysis showed that MSC-derived exosomes contained 857 proteins related to biological processes such as communication, movement, and inflammation [66]. Otero-Ortega et al. [67] investigated the use of MSC-EVs in a subcortical ischemic stroke model. Through proteomic analysis, it was determined to contain 2416 proteins related to brain function repair. Eirin et al. [68] used proteomics to characterize the cargo of a pork-derived MSC-EV and found that it contained 4.,937 different proteins. In a subsequent differential expression analysis, they found that MSC-EVs selectively enriched 128 proteins compared to MSCs. These proteins have specific biological characteristics and are closely related to their mediated tissue regeneration functions, such as angiogenesis, coagulation, and apoptosis. By comparing MSC-EVs with some proteins in the immune process in detail, Mardpour et al. [69] found that MSC-EVs secrete some chemokine receptors such as IL10, HGF, LIF, etc., which promotes the migration of MSC-EVs to the site of inflammation and inhibits the occurrence of inflammation and autoimmune diseases. In addition to protein cargo, some reports have also confirmed that MSC-derived exosomes contain a genetic cargo of miRNA and mRNA to achieve control of targeted transcription factor activity and regulate the activity of proteins with tissue repair functions [70,71]. These characteristics provide the scientific basis for MSC-derived exosomes to be used as a drug delivery vehicle in disease monitoring and treatment.

### 2.3. Uniqueness of MSC-Derived Exosomes

MSCs have long been known in the field of regenerative medicine for their therapeutic potential of regenerative potency, low immunogenicity, and long half-life. A large number of subsequent studies have confirmed that the regenerative effectiveness of MSCs is due to their paracrine effects. Therefore, MSC-EVs have an advantage as a medical product over complete cells in theory. Exosomes, as the only endosomal origin EV among the three [72], have the most cell-free therapeutic potential due to their biological activity and ability to mediate intercellular communication [73,74]. 

MSC-derived exosomes are loaded with proteins, nucleic acids, lipids, etc., from MSCs and have similar therapeutic effects to MSCs [75]. Compared with exosomes derived from other donor cells, they have higher stability, lower immunogenicity, and can be engineered to enhance therapeutic effect. They have significant potential and wide application prospects in regenerative medicine and the treatment of many diseases [76]. In addition, MSC-derived exosomes have low immunogenicity and the safety of not directly forming tumors compared to traditional whole cell-based therapies [77]. Most notably, MSC-derived exosomes have great potential as a drug delivery vehicle. As a natural transporter, exosomes can cross the blood–brain barrier [78], which means they can deliver drugs to the brain to treat related diseases. Moreover, the double lipid membrane outside the structure makes it possible for exosomes to support both hydrophilic and hydrophobic drugs [79]. The smaller molecular weight also makes MSC-derived exosomes easy to produce, store, and transport [80]. What is exciting is that MSC-derived exosomes as a drug delivery vehicle in the cardiovascular system [81,82], nervous system [83], kidney [84], skin [85], bone [86], and other aspects of the treatment effect have been proven. The excellent diagnostic potential of MSC-derived exosomes seems to have made them one of the most promising weapons to solve the problems of modern medicine.

## 3. Preparation Method of MSC-Derived Exosomes

**Figure 2:**
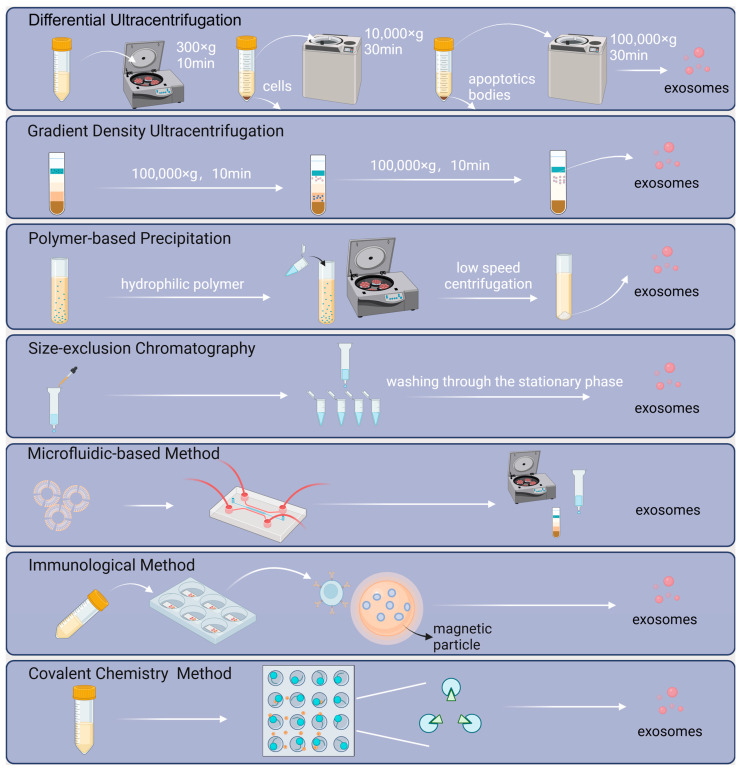
Schematic of preparation for MSC-derived exosomes. Classical separation methods include ultracentrifugation, density gradient centrifugation, polymer-based precipitation, and size-exclusion chromatography. Concurrently, technologies for isolation, exemplified by microfluidics, immunology, and covalent chemistry, are in development.

To advance exosome-related research for potential medical applications, an easy, fast, high-purity separation and preparation method is essential [87]. Based on the size, density, surface proteins, and immunological features of exosomes, researchers proposed a variety of classical techniques (Figure 2), such as differential ultracentrifugation, density gradient centrifugation, polymer-based precipitation, and size-exclusion chromatography [88].

The most commonly and frequently utilized method is ultracentrifugation [89], which can be classified into differential ultracentrifugation and density gradient ultracentrifugation. Accepted as the “gold standard”, differential ultracentrifugation is performed with centrifugal force for multiple cycles of centrifugation to remove cells, cell debris, and apoptotic bodies sequentially, while density gradient ultracentrifugation concentrates on differences between the density of the particles and that of inert media. A certain relative centrifugal force can keep different particles in their particular positions in the gradient medium. 

Polymer precipitation is a high-yield, relatively cost-effective, and commercially viable method for isolating exosomes [90]. Based on the ability of polymers to capture water molecules surrounding exosomes and create a hydrophobic microenvironment, exosomes can be separated from the solution [91]. 

In the size exclusion chromatography-based exosome preparation method, a solvent is used as the mobile phase, and a porous packing material (e.g., porous silica gel or porous resin) is flowed through as the stationary phase. Different particles are eluted sequentially according to size [91,92].

Additionally, with new technologies emerging in recent years, the development of comprehensive and integrated strategies combining the advantages of respective techniques remains promising for the future [93]. At present, the novel strategies for exosome preparation are in terms of microfluidics, immunology, and covalent chemistry in three directions (Figure 2).

For instance, according to the literature, Liang et al. [94] developed a double-filtration microfluidic device aimed at isolating exosomes from urine. Based on the label-free isolation method, the device integrates high-throughput features while having advantages in terms of cost, repeatability, and reducing potential contamination, thereby facilitating downstream analysis and applications [94,95]. Microfluidic technology is a field that involves the manipulation and control of extremely small amounts of fluids [96], typically in the range of microliters (10^−6^ L) to picoliters (10^−12^ L), within microscale channels or chambers. This technology enables precise fluid handling at the microscale level, providing benefits such as efficiency, automation, and integration.

Immunological methods for capturing exosomes involve high-affinity antibody–antigen reactions. The discovery of all exosomes sharing similar surface proteins and the development of magnetic nanomaterials opens up more possibilities for this approach. Yufei Yan et al. [82] proposed a magnetic nanoparticle-associated strategy through specific glycan recognition by lectins, achieving target exosome manipulation and isolation. Commercial immunoaffinity reagents (such as the Exosome-Human EpCAM Isolation Reagent) are now available for isolating specific exosome subpopulations.

For the convenience and simplicity of melanoma circulating tumor cells capture (melanoma CTCs, MelCTCs), and hence for the improvement of subsequent targeting accuracy, Ke Kang et al. [97] present an extracellular vesicle camouflage strategy to generate functionalized magnetic vesicles (Fe_3_O_4_@lip/ev) with anti-fouling and active tumor cell targeting properties. In combination with bioorthogonal click chemistry, dibenzocyclooctyl magnetic capsules can be broadly used for the targeting and isolation of CTCs from all metabolic markers of various phenotypes, organ sources, and even biological species. Similarly, to achieve rapid capture of exosomes, the Click Beads concept was proposed by Sun et al. [98]. The labeling of exosomes was performed by inserting the lipid substrate of DSPE-PEG_1000_-TCO into the exosomes‘ membrane. The TCO-labeled exosomes were captured on Click Beads by bioorthogonal click chemistry between TCO and Tz motifs. Exosomes isolated on Click Beads were collected in a cuvette by centrifugation.

## 4. Methods for Loading Drugs into Exosomes

Given their high biocompatibility, bilayer, and compartment structures, exosomes have the ability to load various pharmaceutical ingredients, including nuclear acids [99], proteins [100], and small molecules [101]. In general terms, the common exosome drug loading methods are divided into endogenous and exogenous.

The endogenous loading method is primarily through changing the biogenesis process of exosomes, pre-loading drugs into intraluminal space or onto the membrane so that donor cells can produce drug-loaded exosomes directly [102]. For example, Masaki Morishita et al. [103] incubated streptavidin and cadherin exosomes (1 mg of protein) with 100 pmol of biotinylated CpG DNA for 10 min at room temperature to present an exosome-based tumor antigens-adjuvant co-delivery system. Lin et al. [104,105] introduced safer and simpler CRISPR/Cas9 non-viral pharmaceutical ingredient deliveries based on exosomes, aiming to attenuate side effects such as immunogenicity and carcinogenicity caused by viral systems in cancer therapy. Ma et al. [88] developed an endogenous loading strategy by pre-transfecting plasmids containing target genes into human adipose-derived MSCs (hAd-MSCs) to produce therapeutic exosomes for enhanced angiogenic–osteogenic regeneration. Vascular endothelial growth factor A (VEGF-A) and bone morphogenetic protein 2 (BMP-2) mRNAs could be transcribed from the above plasmid DNAs and then loaded in ILVs inside MVBs. In addition, the secretion of these therapeutic MSC-derived exosomes could be enhanced via mTORC1-autophagic activities activating by track-etched membrane-based nanoelectroporation, and further separation and purification de-pended on tangential flow filtration (TFF) and size exclusion chromatography (SEC).

In comparison, exogenous loading methods are more widely utilized due to their simplicity of operation, and common strategies include electroporation, sonication, transfection, extrusion microwave-assisted method, saponin-assisted loading, etc. [106]. 

Electroporation is based on the principle that stimulation by an electric field forms holes in the exosome membrane, which enhances permeability and allows the entry of drugs by diffusion [107]. This method is suitable for loading large nucleotides. In this way, Liang et al. [108] successfully loaded an miR-21 inhibitor and chemotherapy drugs into exosomes. At a voltage of 1000 V and a time constant of 10 ms, the loading rate of miR-21 inhibitors and 5-FU in exosomes reached approximately 0.5% and 3.1%, respectively. Mukhopadhya et al. [109] mixed 125 µg MSC-EVs and 250 µg Doxorubicin (with a mass ratio of 1:2), and 100 μL of the mixture was absorbed into the electroporation transfection system Neon™ and proceeded electroporation with 1500 V. Detecting with HPLC and comparing with non-lysed samples, 90.1 ± 12 µg Doxorubicin proved to be successfully loaded into MSC-EVs.

Sonication is a technique that uses probe sonication to create temporary pores in the exosome membrane. This process allows small hydrophilic molecules to diffuse into the exosomes. The exosome membrane can typically be restored by incubating it at 37 °C for 1 h [110]. Gao et al. [111] utilized the sonication strategy to design M2-type primary peritoneal macrophage exosomes as a drug carrier for berberine. Exosomes measuring 125 ± 12 nm were loaded by ultrasound, achieving a drug loading of 17.13 ± 1.64%. The release experiments demonstrated that loaded drugs had a slow release effect, with a cumulative release of 71.44 ± 2.86% within 48 h.

Transfection is a method using transfection reagents to encapsulate drugs [30]. By transfecting adipose MSCs, Liu et al. [112] generated exosomes containing miR-320d mimics.

The extrusion method involves loading a mixture of exosomes and drugs into a lipid extruder with a porous membrane (aperture: 100–400 nm) to load the drug. The method has a high drug-loading efficiency and produces uniform exosome sizes. Extruded exosomes were isolated from human umbilical cord MSCs (hUC MSCs), and exosomes were identified following ultracentrifugation. Zhang et al. [113] conducted a proteomic analysis, with the results showing that 2315 proteins were identified.

Microwave radiation (MR) is a non-ionizing electromagnetic field with frequencies between 300 MHz and 300 GHz. It can increase cell permeability by both thermal and non-thermal effects. Luisa Fernanda Briones-M’arquez et al. [114] employed high-performance liquid chromatography (HPLC), X-ray diffraction (XRD), and flow cytometry to investigate the impact of exposure time, loading method, and type of nutraceutical on loading efficiency, indicating that MR is a potentially promising method.

Cao et al. [115] demonstrated that saponin, as a potent membrane-penetrating agent, allows the formation of complexes with membrane cholesterol, thereby creating pores as a means of increasing the permeability of cell membranes. Using saponin infiltration, Oshchepkova et al. [116] successfully loaded synthetic single-stranded oligonucleotides into natural exosomes and two artificial mimics derived from primary human endometrial MSCs.

Currently, the clinical application of exosomes as drug carriers still faces many challenges, and there are advantages and disadvantages between different approaches. Electroporation is less safe in industrial production and not suitable for large-scale applications [107]. Transfection requires extra components leading to relatively high toxicity, thus limited for safety considerations [117]. Transfection agents may have an effect on gene expression in exosomes produced by donor cells, potentially impacting the nucleic acid drugs transported by exosomes [118]. Continuing to optimize the drug-loading technology to maximize the stable encapsulation of bioactive substances and improve the drug delivery capacity through endogenous and exogenous methods will be the future research direction.

## 5. Application of MSC-Derived Exosomes as Drug Delivery Systems

**Figure 3:**
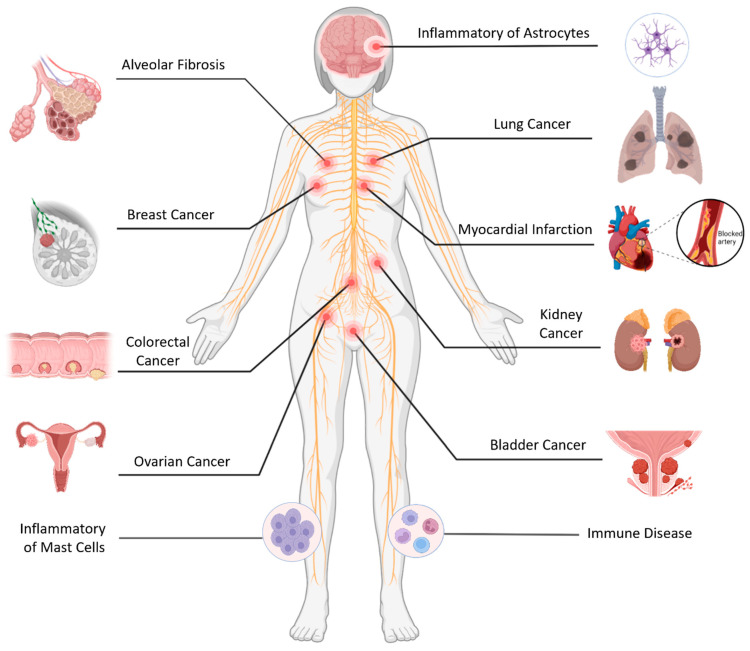
MSC-derived exosomes as drug delivery systems from different tissues can be applied for disease therapy such as inflammatory disease, cancer, immune disease, ischemic disease, and fibrotic disease.

A drug delivery system refers to the device or technology that can deliver drugs to the target area in time or space as expected, helping to control the release and distribution of drugs in the body, reducing the cost of drug delivery and the harm of drugs to the human body. MSC-derived exosomes have similar therapeutic functions to MSCs, and there are some specific markers of MSC-derived exosomes, such as CD29, CD90, CD73, CD44, etc. This makes MSC-derived exosomes a very superior drug delivery vehicle for disease therapy (Figure 3). However, despite the potential of therapies derived from MSC exosomes in the treatment of diseases, their application in clinical settings remains challenging due to issues such as immune compatibility, unstable action of contents, and the spread of exosomes to other tissues [119].

### 5.1. Inflammatory Disease

Inflammatory diseases include infectious diseases and non-infectious inflammatory diseases. Infectious diseases are caused by bacterial, viral, fungal, or parasitic infections, such as pneumonia and influenza. Non-infectious inflammatory diseases are caused by immune responses or other causes, such as rheumatoid arthritis, inflammatory bowel disease, etc. With the in-depth study of MSC-derived exosomes, it is gradually found that MSC-derived exosome-miRNAs play a key role in promoting wound healing. They have been stated to regulate inflammatory responses, promote epidermal cell proliferation and migration, stimulate fibroblast proliferation and collagen synthesis, and regulate extracellular matrix formation [120]. Due to these functions, MSC-derived exosome-miRNAs have great potential in promoting the healing of inflammatory diseases.

Xian et al. [121] studied the anti-inflammatory effect of MSC-derived exosomes in the treatment of stroke, epilepsy, traumatic brain injury, and other neurological diseases through experiments. Experimental results have stated that MSC-derived exosomes can bind to hippocampal astrocytes both in vitro and in vivo and can reduce reactive astrocyte hyperplasia and inflammation. In addition, researchers also found that MSC-derived exosomes improved lipopolysaccharide-induced mitochondrial dysfunction and calcium signaling abnormalities, as well as status epilepticus-induced memory and learning barriers in mice.

**Figure 4:**
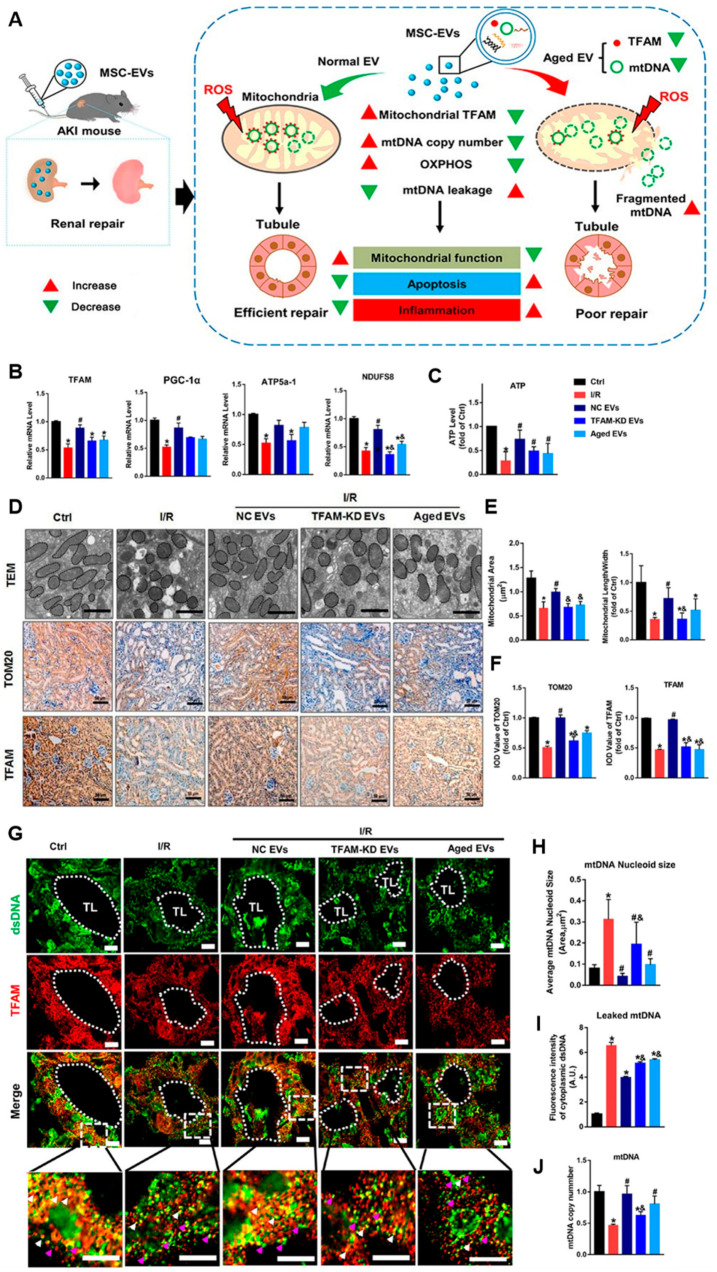
MSC-EVs attenuated renal mitochondrial and mtDNA damage after acute kidney injury (AKI) [122]. (**A**) MSC-derived exosomes attenuate mitochondrial damage and inflammation by stabilizing mitochondrial DNA. (**B**) *TFAM*, *PGC-1α*, *NDUFS8*, and *ATP5a1* mRNA levels in the mouse kidneys on day 3 after AKI (n = 6; * *p* < 0.05 vs. Ctrl group; # *p* < 0.05 vs. I/R group; & *p* < 0.05 vs. NC EV group). (**C**) ATP production in the mouse kidneys (n = 6; * *p* < 0.05 vs. Ctrl group; # *p* < 0.05 vs. I/R group). (**D**) Representative TEM images (scale bar = 2 μm) and micrographs of TOM20 and TFAM IHC staining in kidneys of mice (scale bar = 50 μm). (**E**) Mitochondrial areas and mitochondrial length/width ratio in TEM images (* *p* < 0.05 vs. Ctrl group; # *p* < 0.05 vs. I/R group; & *p* < 0.05 vs. NC EV group). (**F**) Quantification of TOM20 and TFAM protein expression in the kidneys detected using IHC staining (n = 6; * *p* < 0.05 vs. Ctrl group; # *p* < 0.05 vs. I/R group; & *p* < 0.05 vs. NC EV group). (**G**) Representative micrographs of TFAM and dsDNA costaining in mouse renal tubules on day 3 after surgery. The mice with I/R injury received intravenous EV injections (∼6.96 × 10^10^ particles/mouse) (scale bar = 20 μm). Renal tubular lumen (TL), normal mtDNA nucleoid (white arrowheads), and leaked mtDNA (dsDNA that was not colocalized with TFAM, pink arrowheads) were labeled. (**H**) The average size of mtDNA nucleoids in the renal tubules detected using IF staining (n = 10; * *p* < 0.05 vs. Ctrl group; # *p* < 0.05 vs. I/R group; & *p* < 0.05 vs. NC EV group). (**I**) Quantification of the leaked mtDNA intensity (n = 10; * *p* < 0.05 vs. Ctrl group; & *p* < 0.05 vs. NC EV group). (**J**) mtDNA copy number in kidneys of mice (* *p* < 0.05 vs. Ctrl group; # *p* < 0.05 vs. I/R group; & *p* < 0.05 vs. NC EV group).

In the study by Zhao et al. [122], they found that MSC-derived exosomes could attenuate mitochondrial damage and inflammation by stabilizing mitochondrial DNA (Figure 4). These studies reveal that MSC-derived exosomes, as drug delivery in disease therapy, have great potential in treating inflammatory responses triggered by astrocyte hyperplasia and mitochondrial lesions. This also suggests that MSC-derived exosomes have great potential as a nanotherapeutic agent for the treatment of neuropathic diseases in which hippocampal astrocytes are altered. 

Cho et al. [123] have found that tonsillar-MSC-derived exosomes can effectively reduce the inflammatory response of mast cells. On this basis, by analyzing mast cell transcription using DNA microarrays, the researchers concluded that tonsillar-MSC-derived exosomes could regulate the normal physiological-related genes of human mast cells, thereby affecting mast cell inflammation.

Through genetic engineering and photogenetic techniques, Zhao et al. [124] made endothelial NO synthase (eNOS) spontaneously loaded into human umbilical cord MSC-derived exosomes under blue light to obtain UCMSC-exo/eNOS to target the treatment of wounds. The experiment revealed that UCMSC-exo/eNOS significantly improves the wound healing rate of diabetic mice, enhances angiogenesis and matrix remodeling, improves the inflammatory characteristics of the wound site, and regulates the associated immune microenvironment, thus significantly promoting tissue repair, providing a new treatment strategy for promoting the angiogenesis and tissue repair of chronic diabetic wounds. 

On the tissue regeneration potential of MSC-derived exosomes, Byun et al. [125] studied the role of adipose-MSC-derived exosomes (AD-MSC-derived exosomes) from adipose tissue on a mouse model of muscle deficiency. The mouse model was created by the researchers using biopsy perforations in the quadriceps muscle of the hind limb. Experiments have shown that the shape and size of muscle bundles in mice treated with AD-MSC-derived exosomes are relatively intact. Immunohistochemical staining showed a higher expression of myogenin and myoblast determination protein 1 in AD-MSC-derived-exosome-treated mice. These results suggest that AD-MSC-derived exosomes have therapeutic potential for skeletal muscle regeneration.

### 5.2. Cancer

Neoplastic diseases, also known as malignant tumors or cancers, are diseases caused by abnormal cell proliferation and out of control, such as breast cancer, lung cancer, etc. Although modern medicine has made considerable advances in oncology, cancer remains one of the deadliest diseases worldwide. The resistance mechanisms acquired by cancer cells and inefficient drug delivery limit the therapeutic effectiveness of existing chemotherapy drugs. However, recent studies have revealed that nano-drug carriers (NDCs) can break through these limitations [126]. In this sense, exosomes are potential candidates for NDCs. The MSC-derived exosome, as a carrier of information transfer between cells, plays an important role in the formation of the initial structure and site metastasis of tumors. Moreover, due to its therapeutic activity, the body has a low immune rejection reaction to it. Therefore, MSC-derived exosomes have become very superior NDCs for tumor therapy [127].

Huang et al. [128] investigated the effect and molecular mechanism of Platelet-Derived Growth Factor D (PDGFD) carried by MSC-derived exosomes on the growth and metastasis of lung tumors in vitro and in vivo. By comparing PDGFD-carrying MSC-derived exosomes with those treated with anti-PDGFD antibodies, the researchers concluded that MSC-derived exosomes promote lung tumor migration through PDGFD. After Western blot analysis, the researchers found that epithelial-mesenchymal transition (EMT) and PI3K signals were significantly weakened in the anti-PDGFD group.

Jeong et al. [129] studied the anti-tumor effect of canine MSC-derived exosomes in canine mammary tumor cells REM134 and revealed through controlled experiments that the expression levels of MMP-3, IL-1β, IL-6, and TNF-α were down-regulated in the MSC-derived exosome group in REM134. 

By studying the effect of Doxorubicin (Dox) on MSC-derived exosomes in breast cancer cells (bc), Luo et al. [130] found that the exosome secreted by Dox-treated MSCs (Dt-MSC-derived exosomes) silenced the expression of miR-21-5p, enhanced the drug resistance gene S100A6 in bc, and induced a higher degree of Dox resistance. These results revealed the role of MSC-derived exosomes in drug resistance in tumor therapy. 

**Figure 5:**
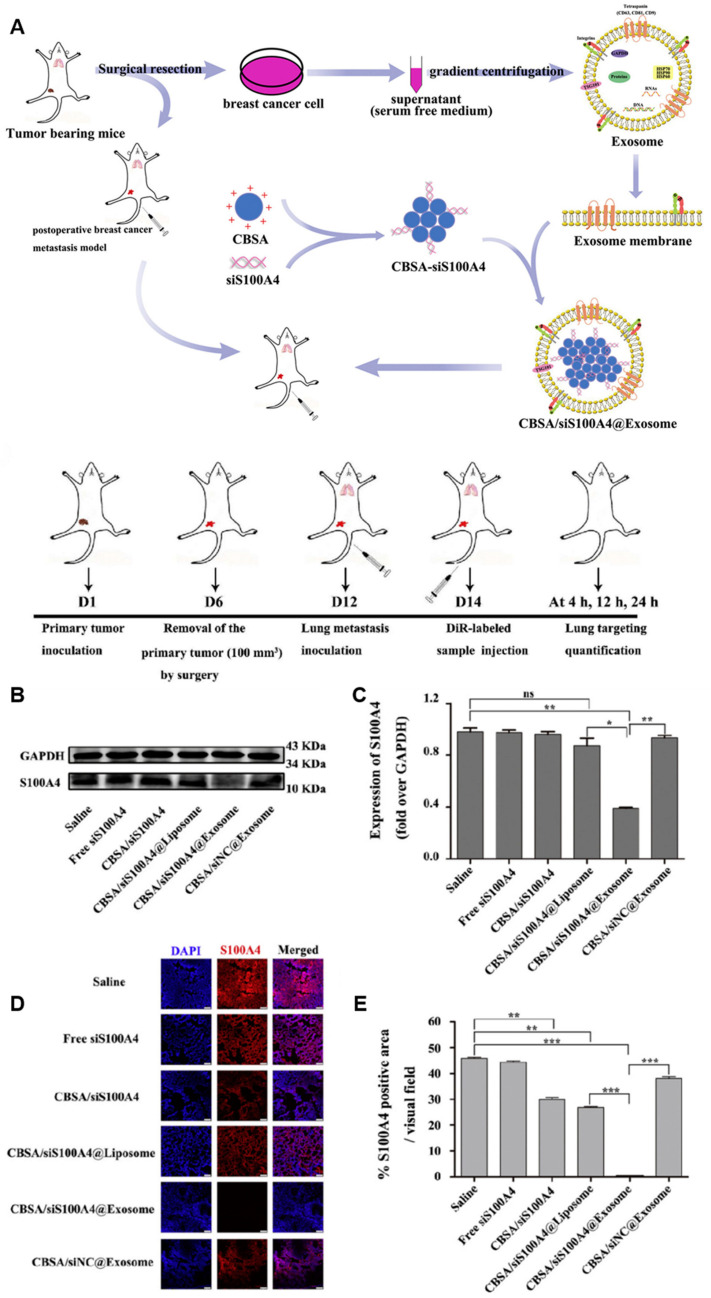
Schematic representation of postoperative lung metastasis model and drug therapy and S100A4 expression in the lung post-treatment [131]. (**A**) Schematic illustration of exosome-mediated siRNA delivery to suppress postoperative breast cancer metastasis. (**B**) Expression of S100A4 in the lung determined by Western blot analysis after treatment with saline, free siS100A4, CBSA/siS100A4, CBSA/siS100A4@Liposome, CBSA/siS100A4@Exosome, and CBSA/siNC@Exosome. (**C**) S100A4/GAPDH values in the lung tissues of each group; the data represent the mean ± SE (n = 4, * *p* < 0.05, ** *p* < 0.01). (**D**) Immunostaining with anti-S100A4 antibody and Cy3-conjugated secondary antibody (red) showing immunofluorescence images of S100A4 expression in lung tissues from each group. Nuclei were stained with DAPI (blue) and samples were imaged by laser scanning confocal microscopy. Scale bar = 75 μm. (**E**) Quantitative assessment of S100A4 in treated lung tissue. The data represent the mean ± SE (n = 4, ** *p* < 0.01, *** *p* < 0.001).

Zhao et al. [131] loaded S100A4 siRNA (siS100A4) onto cationic bovine serum albumin (CBSA) and coated it with a breast cancer cell-derived exosome membrane to produce pharmaceutical nanoparticles (CBSA/siS100A4@Exosome) that targeted the lungs and silenced the S100A4 expression to avoid metastasis of triple-negative breast cancer (TNBC) to the lungs and inhibit tumorigenesis (Figure 5).

Rezaeian et al. [132] utilized MSC-derived exosomes to treat four different cancer cell lines: ACHN, 5637, LNCaP, and PC3. They represent kidney cancer, bladder cancer, hormone-sensitive prostate cancer, and hormone-refractory prostate cancer, respectively. Researchers used the real-time PCR method and showed that the expression of TP53 was increased in the 5637, LNCaP, and PC3 cell lines, and the expression of BCL2 was decreased. In the PC3 cell line, OPNb and OPNc were higher than P53. VEGF-c was increased in the LNCap cell line. In addition, in the 5637 cell line, the expression of two genes, VEGFa and B.A.X, was also reduced. These changes in the expression of the target genes in ACHN suggest that the MSC-derived exosome has antitumor effects, which are more pronounced in bladder cancer, moderate in prostate cancer, and mild in kidney cancer.

Jahangiri et al. [133] treated colorectal cancer (CRC) cells with MSC-derived exosomes and found that MSC-derived exosomes enriched miRNA-100 (miR-100). Later, CRC cells were treated with MSC-derived exosomes combined with anti-miR-100 and compared with the experimental results of previous MSC-derived exosomes treated alone. It was concluded that MSC-derived exosomes reduce the expression of m-TOR, Cyclin D1, K-RAS, and HK2, and increase the expression of p27 and miR-43, which was achieved through miR-100. It provides a new idea for the treatment of cancer.

Ning et al. [134] found that when the concentration of miR-99b-5p is increased, the proliferation and migration of CRC cells are inhibited, while when it is decreased, the effect is reversed, and human bone marrow-MSC-derived exosomes (BMSC-derived exosomes) carrying miR-99b-5p inhibit the development of CRC cells both in vitro and in vivo. This suggests that MSC-derived exosomes may transfer miR-99b-5p into CRC cells, providing a new target for the treatment of CRC.

MSC-derived exosomes exhibit unique properties in cancer therapy, which makes them ideal tools for delivering therapeutic agents to tumor cells. Tumor is one of the biggest diseases threatening human health, and the exploration of tumor treatment has never stopped. MSC-derived exosomes have therapeutic advantages and are more suitable as targeted carriers for tumor treatment.

### 5.3. Immune Disease

Immune diseases refer to disorders caused by abnormal functioning of the immune system, such as autoimmune diseases (like systemic lupus erythematosus and rheumatoid arthritis), allergic reactions, etc. At present, many researchers have investigated the role of MSCs in the prevention and treatment of allergic asthma. In recent years, the effect of MSC-derived exosomes in many diseases has proven to be a promising alternative to relying on MSCs for treatment.

Ren et al. [135] studied the immunomodulatory effect of MSC-derived exosomes in the mouse model of asthma, built the mouse model of asthma, and then used flow cytometry to track and analyze pulmonary interstitial macrophages (IMs) and alveolar macrophages (AMs). Experimental results have exposed that intranasal administration of MSC-derived exosomes significantly increases IL-10-producing IMs in the lung (possibly originating in the spleen), thereby helping to prevent allergic asthma in mice.

It is important to study the mechanism and regulatory factors of the M1-type polarization of the macrophage for the treatment of systemic lupus erythematosus (SLE). Dou et al. [136] believe that the study of MSC-derived exosomes has a certain influence on M1-type polarization of macrophages, and it is confirmed that MSC-derived exosomes may inhibit M1-type polarization of macrophages by transferring tsRNA-21109. Therefore, tsRNA-21109 may become a new therapeutic target for SLE, providing a new direction for the treatment of immune diseases.

Zhou et al. [137], in their studies on pancreatic ductal adenocarcinoma (PDAC), explored a drug delivery system, constructed from MSC-derived exosomes, electroporation-loaded galectin-9-siRNA, and surf-modified oxaliplatin (OXA), forming an exosome-based dual delivery biological system. A synergistic immune response was achieved in in situ PDAC mice by inducing immunogenic cell death (ICD) to stimulate and interfere with immunosuppression. MSC-derived exosomes, due to their significant homing effect, significantly improve the tumor targeting efficacy, resulting in drug accumulation at the tumor site, and alleviating side effects on normal tissues of the body.

### 5.4. Ischemic Disease

Ischemic diseases are diseases caused by insufficient blood supply to tissues or organs, such as ischemic heart disease and stroke. In recent years, the therapeutic effect of MSC-derived exosomes on ischemic stroke has received extensive attention.

Liu et al. [138] established a mouse model of ischemic brain injury caused by middle cerebral artery occlusion by the thread peg method in vivo and conducted a controlled experiment by simulating ischemic conditions in vitro. The results suggest that MSC-derived exosomes may reduce ischemic brain injury by regulating the IL-33/ST2 signaling pathway. Therefore, MSC-derived exosomes may be a potential treatment for ischemic stroke. 

Zheng et al. [139] studied the therapeutic effect of hemin-MSC-derived exosome pretreatment with hemin on myocardial infarction and explored its potential mechanism. The researchers compared the MCS-derived exosome group with the hemin-MSC-derived exosomes group, and the results showed that MSC-derived exosomes and hemin-MSC-derived exosomes improved cardiomyocyte aging and mitochondrial division in vitro and in vivo, with hemin-MSC-derived exosomes having a better protective effect. 

**Figure 6:**
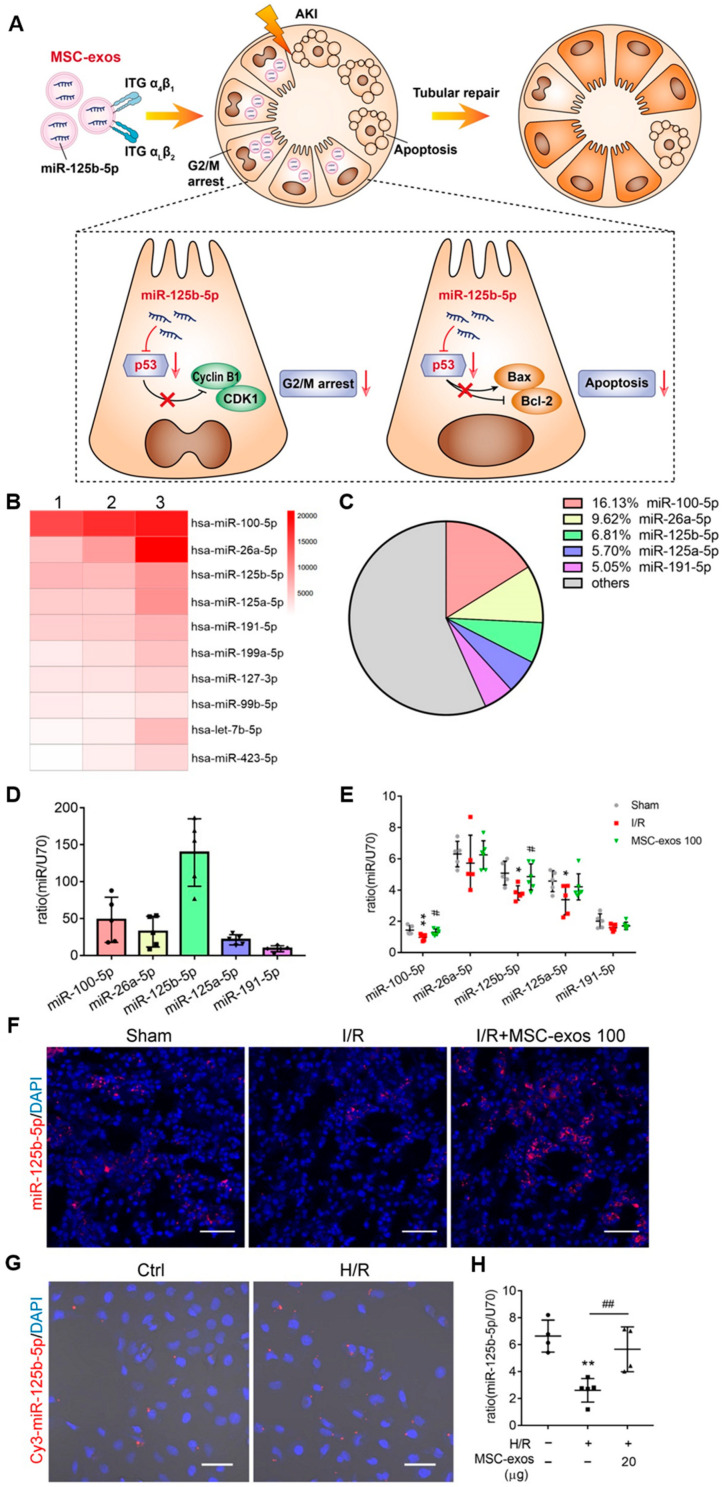
The miR-125b-5p is enriched in MSC-derived exosomes and delivers to TECs [140]. (**A**) In ischemic AKI, the injuries of TECs could lead to cell cycle arrest in the G2/M phase and apoptosis. MSC-derived exosomes targeted the injured kidney especially the proximal tubules due to VLA-4 and LFA-1 mediated adhesive interactions. Moreover, miR-125b-5p was enriched in MSC-derived exosomes and exerted the tubular repair effect via suppressing the expression of p53, which not only up-regulated CDK1 and Cyclin B1 to rescue tubular G2/M arrest but modulated Bcl-2 and Bax to inhibit TEC apoptosis. (**B**) Heat map of the top ten most abundant miRNAs in MSC-exos by miRNA-seq. (**C**) The relative percentage of miRNAs in total miRNA reads. (**D**) RT-PCR analysis of the top five most abundant miRNAs in MSC-derived exosomes (n = 5). (**E**) RT-PCR analysis of the top five miRNAs in MSC-derived-exosome-treated mice renal tissues (n = 5–6). (**F**) FISH analysis of miR-125b-5p in kidney tissues. Scale bars, 50 µm. (**G**) Representative images of Cy3-miR-125b-5p mimic-MSC-derived exosomes internalized by HK-2 cells. Scale bars, 50 µm. (**H**) RT-PCR analysis of miR-125b-5p in HK-2 cells (n = 4–5). Data are presented as mean ± SD, * *p* < 0.05, ** *p* < 0.01 vs. sham group or control group, # *p* < 0.05, ## *p* < 0.01 vs. I/R or H/R group, one-way ANOVA.

Cao et al. [140] found that MSC-derived exosomes could ameliorate ischemic AKI and promote renal tubular repair by targeting the mis-125b-5p/p53 pathway (Figure 6). This study confirms that MSC-derived exosomes could be a promising treatment for AKI.

Ma et al. [141] loaded miR-132, which regulates endothelial cell function during angiogenesis, into MSC-derived exosomes by electroporation. MSC-derived exosomes, as excellent vehicles, effectively solve the difficult problem of miR-132 delivery in vivo. The findings reveal that miR-132 via MSC-derived exosomes targets gene RASA1, reducing its expression and thus promoting angiogenesis, which has potential in ischemic diseases.

### 5.5. Fibrotic Disease

Fibrotic disease is usually manifested as the proliferation and deposition of fibrous tissues in tissues or organs, such as liver cirrhosis and pulmonary fibrosis. Since it was revealed that MSC-derived exosomes can be used as an advantageous drug delivery carrier, many researchers have linked it with fibrotic disease to explore the mechanism.

Zhang et al. [142] explored the protective mechanism of MSC-derived exosomes on experimental pulmonary hypertension (PH). The in vivo and in vitro experimental results uncovered that MSC-derived exosomes could significantly reduce right ventricular systolic blood pressure (RVSP) and right ventricular hypertrophy index in vivo by regulating the Wnt5a/BMP signaling pathway and alleviating pulmonary vascular remodeling and pulmonary fibrosis.

By constructing a mouse model of pulmonary hypertension, Ge et al. [143] also demonstrated that MSC-derived exosomes can significantly reduce right ventricular systolic blood pressure (RVSP) and RVHI and inhibit pulmonary vascular remodeling and the endothelium-mesenchymal transformation (EndMT) process, providing a reliable basis for exploring new methods to treat PH.

Liu et al. [144] investigated the mechanism by which MSC-derived exosomes inhibit neointimal hyperplasia in a rat model of carotid balloon injury. Researchers found that endothelial cells could absorb MSC-derived exosomes, and the expression levels of CD31 and vWF in mice injected with MSC-derived exosomes increased, while the expression level of α-sma decreased.

## 6. Potential Risks of MSC-Derived Exosomes as Drug Delivery Vehicles

As a drug delivery vehicle with great potential, MSC-derived exosomes are attracting widespread attention. These tiny vesicles, which contain bioactive molecules such as miRNA, proteins, and growth factors, are an important way for cells to complete communication. Therefore, they are considered to be drug delivery vehicles and have the ability to treat and diagnose a variety of diseases. However, while the potential is enormous, we still need to recognize the potential risks and challenges of MSC-derived exosomes as drug delivery vehicles.

Suchorska and Lach’s study [145] found that the number of exosomes in cancer patients was much higher than that in healthy controls and confirmed that exosomes perform cell communication functions that make them play a key role in the development and angiogenesis of various types of cancer. As one of the main cargos carried by exosomes, miRNA has become a core participant in shaping tumor micro-environment (TME) in the tumor microenvironment by being able to regulate their expression levels in different cells [146]. Therefore, miRNAs have attracted much attention in cancer research, as they can be used as inhibitors or enhancers of key regulatory proteins and can also directly participate in the transcription and translation of some important genes. It has been widely reported over the past decade that MSC-derived exosomes will exhibit different effects on cancer progression depending on the miRNA species they contain. Therefore, when MSC-derived exosomes are used as drug delivery vehicles, they may lead to the worsening of the patient’s condition. Vallabhaneni et al. [147] pointed out that MSCs under stress will secrete exosomes loaded with tumor-supportive miRNAs, promoting the proliferation and metastasis of breast cancer cells through the paracrine and endocrine mechanisms. The miRNAs involved in cell survival and proliferation, such as miR-21 and miR-34a, are enriched in these exosomes. In the study of Zhou et al. [148], MSC-derived exosomes can also induce and promote epithelial-mesenchymal transformation of cells through the ERK pathway, thus greatly improving the invasion and migration potential of breast cancer cells and promoting the development and metastasis of malignant tumors. 

Apart from promoting tumor invasion and metastasis, the miRNA cargo carried by MSC-derived exosomes can also inhibit the occurrence and development of tumors. For example, MSC-derived exosomes, which carry miR-144-3p, can block and inhibit the development of cervical cancer and promote cancer cell apoptosis by targeting CEP55 [149]. In the study of Xu et al. [150], exosomes secreted by bone marrow-derived MSCs (BM-MSCs) contain an overexpression of miR-16-5p, which can target the down-regulation of ITGA2 and thus stimulate colon cancer cells to apoptosis. There is also a more significant example. In the study of Roccaro et al. [151], the miRNA content of multiple myeloma (MM) BM-MSC-derived exosomes was different from that of normal cell-derived exosomes, mainly due to the lower content of the tumor suppressor gene miR-15a loaded in the former. In addition, there was a large difference in protein composition between the two, with MM BM-MSC-derived exosomes exhibiting higher levels of cancer-causing proteins. It has also been demonstrated that multiple myeloma BM-MSC-derived exosomes promote multiple myeloma, while normal cell-derived exosomes inhibit the development of the disease. These inconsistent results indicate that both the culture conditions of MSCs and the cell origin of exosomes may affect the overall characteristics of these tiny vesicles. When MSCs are subjected to external stressors, such as hypoxia, exposure to inflammatory cytokines, or mechanical stress, the composition and functionality of the EVs they secrete may be affected. Under these stress conditions, MSCs may release exosomes enriched with specific proteins, RNA molecules, or metabolites that could potentially promote tumor growth or metastasis [152]. For instance, growth factors or cytokines within the MSC-derived exosomes might be harnessed by cancer cells to enhance their proliferation, invasion, or evasion of immune surveillance [153]. So, the production and application of exosomes are facing the challenge of standardization [154]. Further research and development of standardized conditions for an MSC culture will help ensure consistency and high quality of their exosome products.

In addition to the necessity of exploring the culture conditions of MSCs, the production of exosome separation, storage, encapsulation process for carrying cargo, selection of delivery targets, and delivery mode is indispensable for the development of MSC-derived exosomes as a disease therapy nanocarrier. Firstly, the targeting of MSC-derived exosomes is highly dependent on the targeted modification, so the degree of understanding of the development of the disease will affect the therapeutic effect [155]. For some diseases with unclear pathogenesis, such as Parkinson’s disease and Alzheimer’s disease, in order to improve the targeting of drug delivery, researchers may need to spend a lot of energy exploring its pathogenesis and finding more accurate and optimized targets or delivery methods. Secondly, low yield and inefficiency are some of the main reasons that prevent exosome-based cell-free therapies from entering clinical practice [156]. It is worth celebrating that bioreactors capable of a 3D culture of MSCs have been developed, such as multi-layer cell culture bottles, hollow fiber bioreactors, stirred tank bioreactors, and spherical aggregates of MSCs [157]. Among them, closed hollow fiber bioreactors [158], porous scaffolds with high surface area to volume ratio, and packed bed plasma immersion ion implantation (PBP13) technology [159] both maintain the original cellular origin characteristics of exosomes with promising results. Thirdly, the diversity of exosomes in size, content, and surface markers makes isolation challenging. Commonly used exosome separation and purification techniques are generally based on their size, surface charge, or immunoaffinity differences, such as ultracentrifugation, size exclusion chromatography, chromatography, etc. However, each approach has its own advantages and disadvantages, and there is no one-size-fits-all approach to choose from. Last but not least, although numerous studies in laboratory and animal models have shown that MSC-derived exosomes have unlimited potential for disease diagnosis and treatment, the exact function of exosomes is not yet clear, so its efficacy and safety in clinical practice still need to be further verified. The lack of clinical data suggests that MSC-derived exosome therapy still has a long way to go. Although MSC-derived exosomes have great prospects as drug delivery vehicles, there are still many potential risks if they are truly realized as a medical means.

The existing clinical cases include the randomized, single-blind, placebo-controlled phase I clinical study on the safety and effectiveness of human umbilical cord MSC-derived exosome nebulizing inhalation in the treatment of pulmonary fibrosis by Chang et al. [160], Tsinghua University, in 2023. However, the number of clinical cases of MSC-derived exosome therapy is small, and the clinical effect is unstable, which still needs further research.

## 7. Conclusions and Future Perspectives 

In this review, the structure, characteristics, preparation methods, and application of MSC-derived exosomes as drug delivery systems in the treatment of diseases are summarized. MSC-derived exosomes have shown great potential and broad prospects as drug delivery vehicles in the field of disease treatment. More and more researchers have devoted themselves to this field, confirming the potential advantages of MSC-derived exosomes as a targeted drug delivery system and elucidating the diversity of their mechanisms. Its special immunomodulatory effects, tissue localization, repair promotion, biocompatibility, and other excellent characteristics make it an ideal candidate for the construction of drug delivery systems. In the treatment of a variety of diseases, such as cardiovascular diseases, nervous system diseases, and cancer, MSC-derived exosomes have shown various application values. However, while MSC-derived exosome therapies hold promise in disease diagnosis and treatment, their application in clinical settings remains challenging due to issues such as immune compatibility and cell stability. Further clinical studies and technical improvements are needed to ensure the safety and efficacy of MSC-derived exosomes. However, further clinical studies and technical improvements are needed to ensure the safety and efficacy of MSC-derived exosomes. At present, the challenges of MSC-derived exosomes as drug delivery vehicles include the optimization of production and purification technology, artificially modifying methods to improve stability, and the improvement of storage and delivery technology. With the deepening of scientific research and the continuous progress of technology, it is believed that MSC-derived exosomes will bring more surprises to the treatment of diseases in the future and become an indispensable and important tool in clinical practice.

## Figures and Tables

**Table 1:** Molecules involved in EV formation and their effects.

Molecules	Type	Effects	Ref.
ESCRT-0 (HRS, HGS, STAM1, VPS28)	complex	Binding and sequestering ubiquitinated cargo through ubiquitin-binding motifs	[37,38]
ESCRT-I (TSG101, VPS37A)	complex	Sorting ubiquitinated cargo into ILVs of MVEs; participating membrane budding into the lumen of the MVBs	[38,39]
ESCRT-II(SNF8, VPS25, VPS36)	complex	Sorting and sequestering ubiquitinated cargo proteins; participating membrane budding with ESCRT-I; modulating the assembly of ESCRT-III helices	[39,40]
ESCRT-III (CHMPs)	complex	Driving membrane neck constriction on MVBs during ILV formation with joint effect of Vps4	[41]
Rab5	GTPase	Participating in endosome fusion to form ESEs	[42]
Rab7	GTPase	Mediating trafficking LSEs	[42]
Rab9	GTPase	Participating in assembly of cargo coats and vesicle budding	[43]
Rab11	GTPase	Docking/tethering of MVBs and promoting Ca^2+^-dependent homotypic fusion process	[44]
Rab27a	GTPase	Regulating the size of MVBs	[45]
Rab27b	GTPase	Functioning in docking and fusion with PM against redistributing MVBs to perinuclear region	[45]
* Rab31	GTPase	Driving ILV formation by binding with flotillin proteins to make EGFR enter MVEs; suppressing MVE degradation	[46]
Rab35	GTPase	Regulating PIP_2_ levels of PM; docking/tethering MVBs	[47]
Rab39	GTPase	Interacting with effector UACA, recruiting Lyspersin to mediate basolateral exosome release	[48]
RAL(RAL-1, RalA, RalB)	GTPase	Driving the fusion between MVBs and PM	[49]
TBC1D10A-C	GTPase-activating protein	Acting on Rab35 to regulate exosome secretion	[50]
Ca^2+^	ions	Acting in homotypic fusion	[44]
SNAREs (syntaxin-4, syntaxin-5, SNAP-23, and VAMP-7)	soluble *N*-ethylmaleimide-sensitive factor attachment protein receptors	Driving the fusion between MVBs and PM	[49,51]
ALIX	scaffold proteins	Interacting with ESCRT-I (subunit TSG101) and ESCRT-III (subunit CHMP4) and participating in cargo sorting and ILV formation	[52]
Heparanase	/	Regulating the syndecan-syntenin-ALIX pathway through cleaving heparan sulfate chains on syndecans, thus facilitating endosomal membrane budding and exosome formation	[53]
Syntenin	membrane scaffold proteins	Interacting with ALIX and contributing to intraluminal budding of endosomal membranes	[54]
Syndecan	membrane scaffold proteins	Recruiting syntenin-ALIX and facilitating membrane budding to form ILVs and exosomes through the syndecan-syntenin-ALIX pathway	[54]
ARF6 and PLD2	/	Controlling the budding of ILVs into MVBs through ALIX–syntenin	[55]
DGKα and PKD1/2	/	Regulating MVB maturation and polarized traffic	[56]
CD81, CD63, CD9	tetraspanins; exosome cargo proteins	Facilitating the trafficking and oligomerization of other membrane proteins	[57]
PE, PS, PA, and lysophospholipid	phospholipids	Promoting exosome biogenesis	[58]
KIBRA	scaffolding protein	Preventing Rab27a from being ubiquitinated and regulating exosome secretion	[59]
* Neutral sphingomyelinase 2 (nSMase2)	sphingomyelinase	Producing ceramide to achieve ESCRT-independent budding machinery	[60]
* Ceramide	/	Improving membrane curvature and regulating the abundance of other lipids, playing a key role in ESCRT-independent budding machinery	[60]
* LAMP2A	membrane protein	Loading proteins (such as HIF1A) into exosomes	[61]

The abbreviations above are explained as follows: PM: plasma membrane; ESEs: early sorting endosomes; LSEs: late sorting endosomes; MVBs: multivesicular bodies; ILVs: intraluminal vesicles; ESCRT: endosomal sorting complex required for transport; ALIX: apoptosis-linked gene 2-interacting protein X; PE: phosphatidylethanolamine PS: phosphatidylserine; PA: phosphatidic acid; EGFR: epidermal growth factor receptor; SNAREs: soluble *N*-ethylmaleimide-sensitive factor attachment protein receptors; ARF6: small GTPase ADP ribosylation factor 6; PLD2: phospholipase D2; DGKα: diacylglycerol kinase α; PKD: protein kinase D. * annotate the molecular involving ESCRT-independent mechanism.

## References

[1] Stoorvogel W., Kleijmeer M.J., Geuze H.J., Raposo G. (2002). The Biogenesis and Functions of Exosomes. Traffic.

[2] Welsh J.A., Goberdhan D.C.I., O’Driscoll L., Buzas E.I., Blenkiron C., Bussolati B., Cai H., Di Vizio D., Driedonks T.A.P., Erdbrügger U. (2024). Minimal information for studies of extracellular vesicles (MISEV2023): From basic to advanced approaches. J. Extracell. Vesicles.

[3] Liu Y., Wang Y., Lv Q., Li X. (2020). Exosomes: From garbage bins to translational medicine. Int. J. Pharm..

[4] Johnstone R.M., Adam M., Hammond J.R., Orr L., Turbide C. (1987). Vesicle formation during reticulocyte maturation. Association of plasma membrane activities with released vesicles (exosomes). J. Biol. Chem..

[5] Valadi H., Ekström K., Bossios A., Sjöstrand M., Lee J.J., Lötvall J.O. (2007). Exosome-mediated transfer of mRNAs and microRNAs is a novel mechanism of genetic exchange between cells. Nat. Cell Biol..

[6] Brose N. (2014). All Roads Lead to Neuroscience: The 2013 Nobel Prize in Physiology or Medicine. Neuron.

[7] Yimin E., Lu C., Zhu K., Li W., Sun J., Ji P., Meng M., Liu Z., Yu C. (2024). Function and mechanism of exosomes derived from different cells as communication mediators in colorectal cancer metastasis. iScience.

[8] Whiteside T.L. (2017). Exosomes carrying immunoinhibitory proteins and their role in cancer. Clin. Exp. Immunol..

[9] Xu Y., Ma L., Wang Y., Shi C. (2024). Engineering strategies of biomaterial-assisted exosomes for skin wound repair: Latest advances and challenges. Chin. Chem. Lett..

[10] Spees J.L., Olson S.D., Ylostalo J., Lynch P.J., Smith J., Perry A., Peister A., Wang M.Y., Prockop D.J. (2003). Differentiation, cell fusion, and nuclear fusion during ex vivo repair of epithelium by human adult stem cells from bone marrow stroma. Proc. Natl. Acad. Sci. USA.

[11] Liu F., Meng F., Yang Z., Wang H., Ren Y., Cai Y., Zhang X. (2024). Exosome-biomimetic nanocarriers for oral drug delivery. Chin. Chem. Lett..

[12] Chavda V.P., Luo G., Bezbaruah R., Kalita T., Sarma A., Deka G., Duo Y., Das B.K., Shah Y., Postwala H. (2024). Unveiling the promise: Exosomes as game-changers in anti-infective therapy. Exploration.

[13] Haynesworth S.E., Baber M.A., Caplan A.I. (1996). Cytokine expression by human marrow-derived mesenchymal progenitor cells in vitro: Effects of dexamethasone and IL-1 alpha. J. Cell. Physiol..

[14] Rak J., Filmus J., Kerbel R.S. (1996). Reciprocal paracrine interactions between tumour cells and endothelial cells: The ‘angiogenesis progression’ hypothesis. Eur. J. Cancer.

[15] Zheng L., Hu B., Zhao D., Liu W., Liu Q., Huang Y., Ruan S. (2024). Recent progresses of exosome–liposome fusions in drug delivery. Chin. Chem. Lett..

[16] Baglio S.R., Pegtel D.M., Baldini N. (2012). Mesenchymal stem cell secreted vesicles provide novel opportunities in (stem) cell-free therapy. Front. Physiol..

[17] Yang J., Li Y., Jiang S., Tian Y., Zhang M., Guo S., Wu P., Li J., Xu L., Li W. (2024). Engineered Brain-targeting Exosome for Reprogramming Immunosuppressive Microenvironment of Glioblastoma. Exploration.

[18] Spelat R., Ferro F., Contessotto P., Warren N.J., Marsico G., Armes S.P., Pandit A. (2020). A worm gel-based 3D model to elucidate the paracrine interaction between multiple myeloma and mesenchymal stem cells. Mater. Today Bio.

[19] Markel T.A., Drucker N.A., Jensen A.R., Olson K.R. (2020). Human Mesenchymal Stem Cell Hydrogen Sulfide Production Critically Impacts the Release of Other Paracrine Mediators After Injury. J. Surg. Res..

[20] Yáñez-Mó M., Siljander P.R., Andreu Z., Zavec A.B., Borràs F.E., Buzas E.I., Buzas K., Casal E., Cappello F., Carvalho J. (2015). Biological properties of extracellular vesicles and their physiological functions. J. Extracell. Vesicles.

[21] Lai R.C., Yeo R.W., Lim S.K. (2015). Mesenchymal stem cell exosomes. Semin. Cell Dev. Biol..

[22] Lai R.C., Arslan F., Lee M.M., Sze N.S., Choo A., Chen T.S., Salto-Tellez M., Timmers L., Lee C.N., El Oakley R.M. (2010). Exosome secreted by MSC reduces myocardial ischemia/reperfusion injury. Stem Cell Res..

[23] Konoshenko M.Y., Lekchnov E.A., Vlassov A.V., Laktionov P.P. (2018). Isolation of Extracellular Vesicles: General Methodologies and Latest Trends. BioMed Res. Int..

[24] Zhang P., Yeo J.C., Lim C.T. (2019). Advances in Technologies for Purification and Enrichment of Extracellular Vesicles. SLAS Technol..

[25] Jiang J., Mei J., Ma Y., Jiang S., Zhang J., Yi S., Feng C., Liu Y., Liu Y. (2022). Tumor Hijacks Macrophages and Microbiota through Extracellular Vesicles. Exploration.

[26] Naseri Z., Oskuee R.K., Jaafari M.R., Forouzandeh Moghadam M. (2018). Exosome-mediated delivery of functionally active miRNA-142-3p inhibitor reduces tumorigenicity of breast cancer in vitro and in vivo. Int. J. Nanomed..

[27] Zhuang M., Chen X., Du D., Shi J., Deng M., Long Q., Yin X., Wang Y., Rao L. (2020). SPION decorated exosome delivery of TNF-α to cancer cell membranes through magnetism. Nanoscale.

[28] Chen T.S., Arslan F., Yin Y., Tan S.S., Lai R.C., Choo A.B.H., Padmanabhan J., Lee C.N., de Kleijn D.P.V., Lim S.K. (2011). Enabling a robust scalable manufacturing process for therapeutic exosomes through oncogenic immortalization of human ESC-derived MSCs. J. Transl. Med..

[29] Chen K.H., Chen C.H., Wallace C.G., Yuen C.M., Kao G.S., Chen Y.L., Shao P.L., Chen Y.L., Chai H.T., Lin K.C. (2016). Intravenous administration of xenogenic adipose-derived mesenchymal stem cells (ADMSC) and ADMSC-derived exosomes markedly reduced brain infarct volume and preserved neurological function in rat after acute ischemic stroke. Oncotarget.

[30] Mao Q., Liang X.L., Zhang C.L., Pang Y.H., Lu Y.X. (2019). LncRNA KLF3-AS1 in human mesenchymal stem cell-derived exosomes ameliorates pyroptosis of cardiomyocytes and myocardial infarction through miR-138-5p/Sirt1 axis. Stem Cell Res. Ther..

[31] Pan W., Chen H., Wang A., Wang F., Zhang X. (2023). Challenges and strategies: Scalable and efficient production of mesenchymal stem cells-derived exosomes for cell-free therapy. Life Sci..

[32] Xu M., Ji J., Jin D., Wu Y., Wu T., Lin R., Zhu S., Jiang F., Ji Y., Bao B. (2023). The biogenesis and secretion of exosomes and multivesicular bodies (MVBs): Intercellular shuttles and implications in human diseases. Genes Dis..

[33] Krylova S.V., Feng D. (2023). The Machinery of Exosomes: Biogenesis, Release, and Uptake. Int. J. Mol. Sci..

[34] Henne W.M., Buchkovich N.J., Emr S.D. (2011). The ESCRT Pathway. Dev. Cell.

[35] Schöneberg J., Lee I.H., Iwasa J.H., Hurley J.H. (2017). Reverse-topology membrane scission by the ESCRT proteins. Nat. Rev. Mol. Cell Biol..

[36] Woodman P.G., Futter C.E. (2008). Multivesicular bodies: Co-ordinated progression to maturity. Curr. Opin. Cell Biol..

[37] Clague M.J., Liu H., Urbe S. (2012). Governance of Endocytic Trafficking and Signaling by Reversible Ubiquitylation. Dev. Cell.

[38] Colombo M., Moita C., van Niel G., Kowal J., Vigneron J., Benaroch P., Manel N., Moita L.F., Thery C., Raposo G. (2013). Analysis of ESCRT functions in exosome biogenesis, composition and secretion highlights the heterogeneity of extracellular vesicles. J. Cell Sci..

[39] Wollert T., Hurley J.H. (2010). Molecular mechanism of multivesicular body biogenesis by ESCRT complexes. Nature.

[40] Henne W.M., Buchkovich N.J., Zhao Y., Emr S.D. (2012). The Endosomal Sorting Complex ESCRT-II Mediates the Assembly and Architecture of ESCRT-III Helices. Cell.

[41] Adell M.A.Y., Vogel G.F., Pakdel M., Mueller M., Lindner H., Hess M.W., Teis D. (2014). Coordinated binding of Vps4 to ESCRT-III drives membrane neck constriction during MVB vesicle formation. J. Cell Biol..

[42] Stenmark H. (2009). Rab GTPases as coordinators of vesicle traffic. Nat. Rev. Mol. Cell Biol..

[43] Carroll K.S., Hanna J., Simon I., Krise J., Barbero P., Pfeffer S.R. (2001). Role of Rab9 GTPase in facilitating receptor recruitment by TIP47. Science.

[44] Savina A., Fader C.M., Damiani M.T., Colombo M.I. (2005). Rab11 promotes docking and fusion of multivesicular bodies in a calcium-dependent manner. Traffic.

[45] Ostrowski M., Carmo N.B., Krumeich S., Fanget I., Raposo G., Savina A., Moita C.F., Schauer K., Hume A.N., Freitas R.P. (2010). Rab27a and Rab27b control different steps of the exosome secretion pathway. Nat. Cell Biol..

[46] Wei D., Zhan W., Gao Y., Huang L., Gong R., Wang W., Zhang R., Wu Y., Gao S., Kang T. (2021). RAB31 marks and controls an ESCRT-independent exosome pathway. Cell Res..

[47] Klinkert K., Echard A. (2016). Rab35 GTPase: A Central Regulator of Phosphoinositides and F-actin in Endocytic Recycling and Beyond. Traffic.

[48] Matsui T., Sakamaki Y., Nakashima S., Fukuda M. (2022). Rab39 and its effector UACA regulate basolateral exosome release from polarized epithelial cells. Cell Rep..

[49] Hyenne V., Apaydin A., Rodriguez D., Spiegelhalter C., Hoff-Yoessle S., Diem M., Tak S., Lefebvre O., Schwab Y., Goetz J.G. (2015). RAL-1 controls multivesicular body biogenesis and exosome secretion. J. Cell Biol..

[50] Hsu C., Morohashi Y., Yoshimura S.-i., Manrique-Hoyos N., Jung S., Lauterbach M.A., Bakhti M., Gronborg M., Moebius W., Rhee J. (2010). Regulation of exosome secretion by Rab35 and its GTPase-activating proteins TBC1D10A-C. J. Cell Biol..

[51] Liu C., Liu D., Wang S., Gan L., Yang X., Ma C. (2023). Identification of the SNARE complex that mediates the fusion of multivesicular bodies with the plasma membrane in exosome secretion. J. Extracell. Vesicles.

[52] Bissig C., Gruenberg J. (2014). ALIX and the multivesicular endosome: ALIX in Wonderland. Trends Cell Biol..

[53] Roucourt B., Meeussen S., Bao J., Zimmermann P., David G. (2015). Heparanase activates the syndecan-syntenin-ALIX exosome pathway. Cell Res..

[54] Baietti M.F., Zhang Z., Mortier E., Melchior A., Degeest G., Geeraerts A., Ivarsson Y., Depoortere F., Coomans C., Vermeiren E. (2012). Syndecan-syntenin-ALIX regulates the biogenesis of exosomes. Nat. Cell Biol..

[55] Ghossoub R., Lembo F., Rubio A., Gaillard C.B., Bouchet J., Vitale N., Slavik J., Machala M., Zimmermann P. (2014). Syntenin-ALIX exosome biogenesis and budding into multivesicular bodies are controlled by ARF6 and PLD2. Nat. Commun..

[56] Mazzeo C., Calvo V., Alonso R., Merida I., Izquierdo M. (2016). Protein kinase D1/2 is involved in the maturation of multivesicular bodies and secretion of exosomes in T and B lymphocytes. Cell Death Differ..

[57] Hemler M.E. (2003). Tetraspanin proteins mediate cellular penetration, invasion, and fusion events and define a novel type of membrane microdomain. Annu. Rev. Cell Dev. Biol..

[58] Pegtel D.M., Gould S.J. (2019). Exosomes. Annu. Rev. Biochem..

[59] Song L., Tang S., Han X., Jiang Z., Dong L., Liu C., Liang X., Dong J., Qiu C., Wang Y. (2019). KIBRA controls exosome secretion via inhibiting the proteasomal degradation of Rab27a. Nat. Commun..

[60] Trajkovic K., Hsu C., Chiantia S., Rajendran L., Wenzel D., Wieland F., Schwille P., Bruegger B., Simons M. (2008). Ceramide triggers budding of exosome vesicles into multivesicular Endosomes. Science.

[61] Ferreira J.V., Soares A.d.R., Ramalho J., Carvalho C.M., Cardoso M.H., Pintado P., Carvalho A.S., Beck H.C., Matthiesen R., Zuzarte M. (2022). LAMP2A regulates the loading of proteins into exosomes. Sci. Adv..

[62] Wubbolts R., Leckie R.S., Veenhuizen P.T.M., Schwarzmann G., Möbius W., Hoernschemeyer J., Slot J.-W., Geuze H.J., Stoorvogel W. (2003). Proteomic and Biochemical Analyses of Human B Cell-derived Exosomes: Potential Implications for Their Function and Multivesicular Body Formation*. J. Biol. Chem..

[63] Simons M., Raposo G. (2009). Exosomes—vesicular carriers for intercellular communication. Curr. Opin. Cell Biol..

[64] Qiu G., Zheng G., Ge M., Wang J., Huang R., Shu Q., Xu J. (2019). Functional proteins of mesenchymal stem cell-derived extracellular vesicles. Stem Cell Res. Ther..

[65] Lai R.C., Lim S.K. (2019). Membrane lipids define small extracellular vesicle subtypes secreted by mesenchymal stromal cells. J. Lipid Res..

[66] Lai R.C., Tan S.S., Yeo R.W., Choo A.B., Reiner A.T., Su Y., Shen Y., Fu Z., Alexander L., Sze S.K. (2016). MSC secretes at least 3 EV types each with a unique permutation of membrane lipid, protein and RNA. J. Extracell. Vesicles.

[67] Otero-Ortega L., Laso-García F., Gómez-de Frutos M.D., Rodríguez-Frutos B., Pascual-Guerra J., Fuentes B., Díez-Tejedor E., Gutiérrez-Fernández M. (2017). White Matter Repair After Extracellular Vesicles Administration in an Experimental Animal Model of Subcortical Stroke. Sci. Rep..

[68] Eirin A., Zhu X.Y., Puranik A.S., Woollard J.R., Tang H., Dasari S., Lerman A., van Wijnen A.J., Lerman L.O. (2016). Comparative proteomic analysis of extracellular vesicles isolated from porcine adipose tissue-derived mesenchymal stem/stromal cells. Sci. Rep..

[69] Mardpour S., Hamidieh A.A., Taleahmad S., Sharifzad F., Taghikhani A., Baharvand H. (2019). Interaction between mesenchymal stromal cell-derived extracellular vesicles and immune cells by distinct protein content. J. Cell. Physiol..

[70] Zou X.Y., Yu Y., Lin S., Zhong L., Sun J., Zhang G., Zhu Y. (2018). Comprehensive miRNA Analysis of Human Umbilical Cord-Derived Mesenchymal Stromal Cells and Extracellular Vesicles. Kidney Blood Press. Res..

[71] Eirin A., Zhu X.Y., Puranik A.S., Woollard J.R., Tang H., Dasari S., Lerman A., van Wijnen A.J., Lerman L.O. (2017). Integrated transcriptomic and proteomic analysis of the molecular cargo of extracellular vesicles derived from porcine adipose tissue-derived mesenchymal stem cells. PLoS ONE.

[72] Tan S.S., Yin Y., Lee T., Lai R.C., Yeo R.W., Zhang B., Choo A., Lim S.K. (2013). Therapeutic MSC exosomes are derived from lipid raft microdomains in the plasma membrane. J. Extracell. Vesicles.

[73] Zhang W., Wang Y., Kong J., Dong M., Duan H., Chen S. (2017). Therapeutic efficacy of neural stem cells originating from umbilical cord-derived mesenchymal stem cells in diabetic retinopathy. Sci. Rep..

[74] Rani S., Ryan A.E., Griffin M.D., Ritter T. (2015). Mesenchymal Stem Cell-derived Extracellular Vesicles: Toward Cell-free Therapeutic Applications. Mol. Ther. J. Am. Soc. Gene Ther..

[75] Asadi K., Amini A., Gholami A. (2022). Mesenchymal stem cell-derived exosomes as a bioinspired nanoscale tool toward next-generation cell-free treatment. J. Drug Deliv. Sci. Technol..

[76] Soler-Botija C., Monguió-Tortajada M., Munizaga-Larroudé M., Gálvez-Montón C., Bayes-Genis A., Roura S. (2022). Mechanisms governing the therapeutic effect of mesenchymal stromal cell-derived extracellular vesicles: A scoping review of preclinical evidence. Biomed. Pharmacother..

[77] Lyu C., Sun H., Sun Z., Liu Y., Wang Q. (2024). Roles of exosomes in immunotherapy for solid cancers. Cell Death Dis..

[78] Khatami S.H., Karami N., Taheri-Anganeh M., Taghvimi S., Tondro G., Khorsand M., Soltani Fard E., Sedighimehr N., Kazemi M., Rahimi Jaberi K. (2023). Exosomes: Promising Delivery Tools for Overcoming Blood-Brain Barrier and Glioblastoma Therapy. Mol. Neurobiol..

[79] Lai R.C., Yeo R.W., Tan K.H., Lim S.K. (2013). Exosomes for drug delivery—A novel application for the mesenchymal stem cell. Biotechnol. Adv..

[80] Kimiz-Gebologlu I., Oncel S.S. (2022). Exosomes: Large-scale production, isolation, drug loading efficiency, and biodistribution and uptake. J. Control. Release.

[81] Zhao Y., Sun X., Cao W., Ma J., Sun L., Qian H., Zhu W., Xu W. (2015). Exosomes Derived from Human Umbilical Cord Mesenchymal Stem Cells Relieve Acute Myocardial Ischemic Injury. Stem Cells Int..

[82] Yan Y., Li R., Chen H., Li Y., Wu M., Wang Z., Yang G. (2023). Magnetic nanoagent assisted deciphering of heterogeneous glycans in extracellular vesicles of varied cellular origins. Biosens. Bioelectron..

[83] Yin T., Liu Y., Ji W., Zhuang J., Chen X., Gong B., Chu J., Liang W., Gao J., Yin Y. (2023). Engineered mesenchymal stem cell-derived extracellular vesicles: A state-of-the-art multifunctional weapon against Alzheimer’s disease. Theranostics.

[84] Jia H., Liu W., Zhang B., Wang J., Wu P., Tandra N., Liang Z., Ji C., Yin L., Hu X. (2018). HucMSC exosomes-delivered 14-3-3ζ enhanced autophagy via modulation of ATG16L in preventing cisplatin-induced acute kidney injury. Am. J. Transl. Res..

[85] Kim S., Lee S.K., Kim H., Kim T.M. (2018). Exosomes Secreted from Induced Pluripotent Stem Cell-Derived Mesenchymal Stem Cells Accelerate Skin Cell Proliferation. Int. J. Mol. Sci..

[86] Guo S., Perets N., Betzer O., Ben-Shaul S., Sheinin A., Michaelevski I., Popovtzer R., Offen D., Levenberg S. (2019). Intranasal Delivery of Mesenchymal Stem Cell Derived Exosomes Loaded with Phosphatase and Tensin Homolog siRNA Repairs Complete Spinal Cord Injury. ACS Nano.

[87] Liu W.-Z., Ma Z.-J., Kang X.-W. (2022). Current status and outlook of advances in exosome isolation. Anal. Bioanal. Chem..

[88] Ma Y., Sun L., Zhang J., Chiang C.L., Pan J., Wang X., Kwak K.J., Li H., Zhao R., Rima X.Y. (2023). Exosomal mRNAs for Angiogenic-Osteogenic Coupled Bone Repair. Adv. Sci..

[89] Coughlan C., Bruce K.D., Burgy O., Boyd T.D., Michel C.R., Garcia-Perez J.E., Adame V., Anton P., Bettcher B.M., Chial H.J. (2020). Exosome Isolation by Ultracentrifugation and Precipitation and Techniques for Downstream Analyses. Curr. Protoc. Cell Biol..

[90] Kamei N., Nishimura H., Matsumoto A., Asano R., Muranaka K., Fujita M., Takeda M., Hashimoto H., Takeda-Morishita M. (2021). Comparative study of commercial protocols for high recovery of high-purity mesenchymal stem cell-derived extracellular vesicle isolation and their efficient labeling with fluorescent dyes. Nanomed. Nanotechnol. Biol. Med..

[91] Gandham S., Su X., Wood J., Nocera A.L., Alli S.C., Milane L., Zimmerman A., Amiji M., Ivanov A.R. (2020). Technologies and Standardization in Research on Extracellular Vesicles. Trends Biotechnol..

[92] Böing A.N., van der Pol E., Grootemaat A.E., Coumans F.A., Sturk A., Nieuwland R. (2014). Single-step isolation of extracellular vesicles by size-exclusion chromatography. J. Extracell. Vesicles.

[93] Gao J., Li A., Hu J., Feng L., Liu L., Shen Z. (2022). Recent developments in isolating methods for exosomes. Front. Bioeng. Biotechnol..

[94] Liang L.-G., Kong M.-Q., Zhou S., Sheng Y.-F., Wang P., Yu T., Inci F., Kuo W.P., Li L.-J., Demirci U. (2017). An integrated double-filtration microfluidic device for isolation, enrichment and quantification of urinary extracellular vesicles for detection of bladder cancer. Sci. Rep..

[95] Hassanpour Tamrin S., Sanati Nezhad A., Sen A. (2021). Label-Free Isolation of Exosomes Using Microfluidic Technologies. ACS Nano.

[96] Tong Z., Yang D., Shen C., Li C., Xu X., Li Q., Wu Z., Ma H., Chen F., Mao H. (2024). Rapid automated extracellular vesicle isolation and miRNA preparation on a cost-effective digital microfluidic platform. Anal. Chim. Acta.

[97] Kang K., Zhang Y., Zhou X., Yu Y., Zhu N., Cheng J., Yi Q., Wu Y. (2023). Hybrid Extracellular Vesicles-Liposomes Camouflaged Magnetic Vesicles Cooperating with Bioorthogonal Click Chemistry for High-Efficient Melanoma Circulating Tumor Cells Enrichment. Adv. Healthc. Mater..

[98] Sun N., Tran B.V., Peng Z., Wang J., Zhang C., Yang P., Zhang T.X., Widjaja J., Zhang R.Y., Xia W. (2022). Coupling Lipid Labeling and Click Chemistry Enables Isolation of Extracellular Vesicles for Noninvasive Detection of Oncogenic Gene Alterations. Adv. Sci..

[99] Wahlgren J., Karlson T.D.L., Brisslert M., Vaziri Sani F., Telemo E., Sunnerhagen P., Valadi H. (2012). Plasma exosomes can deliver exogenous short interfering RNA to monocytes and lymphocytes. Nucleic Acids Res..

[100] Haney M.J., Klyachko N.L., Zhao Y., Gupta R., Plotnikova E.G., He Z., Patel T., Piroyan A., Sokolsky M., Kabanov A.V. (2015). Exosomes as drug delivery vehicles for Parkinson’s disease therapy. J. Control. Release.

[101] Yang T., Martin P., Fogarty B., Brown A., Schurman K., Phipps R., Yin V.P., Lockman P., Bai S. (2015). Exosome Delivered Anticancer Drugs Across the Blood-Brain Barrier for Brain Cancer Therapy in Danio Rerio. Pharm. Res..

[102] Huang C.-C., Kang M., Lu Y., Shirazi S., Diaz J.I., Cooper L.F., Gajendrareddy P., Ravindran S. (2020). Functionally engineered extracellular vesicles improve bone regeneration. Acta Biomater..

[103] Morishita M., Takahashi Y., Matsumoto A., Nishikawa M., Takakura Y. (2016). Exosome-based tumor antigens–adjuvant co-delivery utilizing genetically engineered tumor cell-derived exosomes with immunostimulatory CpG DNA. Biomaterials.

[104] Lin Y.-Q., Feng K.-K., Lu J.-Y., Le J.-Q., Li W.-L., Zhang B.-C., Li C.-L., Song X.-H., Tong L.-W., Shao J.-W. (2023). CRISPR/Cas9-based application for cancer therapy: Challenges and solutions for non-viral delivery. J. Control. Release.

[105] Ye Y., Shi Q., Yang T., Xie F., Zhang X., Xu B., Fang J., Chen J., Zhang Y., Li J. (2022). In Vivo Visualized Tracking of Tumor-Derived Extracellular Vesicles Using CRISPR-Cas9 System. Technol. Cancer Res. Treat..

[106] Batrakova E.V., Kim M.S. (2015). Using exosomes, naturally-equipped nanocarriers, for drug delivery. J. Control. Release.

[107] Pan S., Pei L., Zhang A., Zhang Y., Zhang C., Huang M., Huang Z., Liu B., Wang L., Ma L. (2020). Passion fruit-like exosome-PMA/Au-BSA@Ce6 nanovehicles for real-time fluorescence imaging and enhanced targeted photodynamic therapy with deep penetration and superior retention behavior in tumor. Biomaterials.

[108] Liang G., Zhu Y., Ali D.J., Tian T., Xu H., Si K., Sun B., Chen B., Xiao Z. (2020). Engineered exosomes for targeted co-delivery of miR-21 inhibitor and chemotherapeutics to reverse drug resistance in colon cancer. J. Nanobiotechnol..

[109] Mukhopadhya A., Tsiapalis D., McNamee N., Talbot B., O’Driscoll L. (2023). Doxorubicin Loading into Milk and Mesenchymal Stem Cells’ Extracellular Vesicles as Drug Delivery Vehicles. Pharmaceutics.

[110] Chen Z., Xiong M., Tian J., Song D., Duan S., Zhang L. (2024). Encapsulation and assessment of therapeutic cargo in engineered exosomes: A systematic review. J. Nanobiotechnol..

[111] Gao Z.-S., Zhang C.-J., Xia N., Tian H., Li D.-Y., Lin J.-Q., Mei X.-F., Wu C. (2021). Berberine-loaded M2 macrophage-derived exosomes for spinal cord injury therapy. Acta Biomater..

[112] Liu L., Zhang H., Mao H., Li X., Hu Y. (2019). Exosomal miR-320d derived from adipose tissue-derived MSCs inhibits apoptosis in cardiomyocytes with atrial fibrillation (AF). Artif. Cells Nanomed. Biotechnol..

[113] Zhang Z., Mi T., Jin L., Li M., Zhanghuang C., Wang J., Tan X., Lu H., Shen L., Long C. (2022). Comprehensive proteomic analysis of exosome mimetic vesicles and exosomes derived from human umbilical cord mesenchymal stem cells. Stem Cell Res. Ther..

[114] Briones-Márquez L.F., Navarro-Partida J., Herrera-González A., García-Bon M.A., Martínez-Álvarez I.A., Uribe-Rodríguez D., González-Ortiz L.J., López-Naranjo E.J. (2023). HPLC-UV evaluation of a microwave assisted method as an active drug loading technique for exosome-based drug delivery system. Heliyon.

[115] Cao X.-W., Wang F.-J., Liew O.-W., Lu Y.-Z., Zhao J. (2020). Analysis of Triterpenoid Saponins Reveals Insights into Structural Features Associated with Potent Protein Drug Enhancement Effects. Mol. Pharm..

[116] Oshchepkova A., Neumestova A., Matveeva V., Artemyeva L., Morozova K., Kiseleva E., Zenkova M., Vlassov V. (2019). Cytochalasin-B-Inducible Nanovesicle Mimics of Natural Extracellular Vesicles That Are Capable of Nucleic Acid Transfer. Micromachines.

[117] Momen-Heravi F., Bala S., Bukong T., Szabo G. (2014). Exosome-mediated delivery of functionally active miRNA-155 inhibitor to macrophages. Nanomed. Nanotechnol. Biol. Med..

[118] Ramanathan S., Douglas S.R., Alexander G.M., Shenoda B.B., Barrett J.E., Aradillas E., Sacan A., Ajit S.K. (2019). Exosome microRNA signatures in patients with complex regional pain syndrome undergoing plasma exchange. J. Transl. Med..

[119] Wu D., Tao S., Zhu L., Zhao C., Xu N. (2024). Chitosan Hydrogel Dressing Loaded with Adipose Mesenchymal Stem Cell-Derived Exosomes Promotes Skin Full-Thickness Wound Repair. ACS Appl. Bio Mater..

[120] Ju C., Liu D. (2023). Exosomal microRNAs from Mesenchymal Stem Cells: Novel Therapeutic Effect in Wound Healing. Tissue Eng. Regen. Med..

[121] Xian P., Hei Y., Wang R., Wang T., Yang J., Li J., Di Z., Liu Z., Baskys A., Liu W. (2019). Mesenchymal stem cell-derived exosomes as a nanotherapeutic agent for amelioration of inflammation-induced astrocyte alterations in mice. Theranostics.

[122] Zhao M., Liu S., Wang C., Wang Y., Wan M., Liu F., Gong M., Yuan Y., Chen Y., Cheng J. (2021). Mesenchymal Stem Cell-Derived Extracellular Vesicles Attenuate Mitochondrial Damage and Inflammation by Stabilizing Mitochondrial DNA. ACS Nano.

[123] Cho K.-A., Kwon J., Kim H.J., Woo S.-Y. (2024). Mesenchymal stem cell exosomes differentially regulate gene expression of mast cells. Biochem. Biophys. Res. Commun..

[124] Zhao X., Fu L., Zou H., He Y., Pan Y., Ye L., Huang Y., Fan W., Zhang J., Ma Y. (2023). Optogenetic engineered umbilical cord MSC-derived exosomes for remodeling of the immune microenvironment in diabetic wounds and the promotion of tissue repair. J. Nanobiotechnol..

[125] Byun S.E., Sim C., Chung Y., Kim H.K., Park S., Kim D.K., Cho S., Lee S. (2021). Skeletal Muscle Regeneration by the Exosomes of Adipose Tissue-Derived Mesenchymal Stem Cells. Curr. Issues Mol. Biol..

[126] Araldi R.P., Delvalle D.A., da Costa V.R., Alievi A.L., Teixeira M.R., Pinto J.R.D., Kerkis I. (2023). Exosomes as a Nano-Carrier for Chemotherapeutics: A New Era of Oncology. Cells.

[127] Huang L., Song J., Luo C., Xiong X., Yin M. (2019). Research progress of mesenchymal stem cell-derived exosomes in malignant tumors. Chin. J. Clin. Oncol..

[128] Huang F., Yao Y., Wu J., Yu L., Wu S., Pu X., Xu L., Wang M., Xia L. (2017). Effect of mesenchymal stem cell derived exosomes carrying PDGFD on lung cancer. Int. J. Clin. Exp. Pathol..

[129] Jeong S.Y., Lee S.-A., Gu N.-Y., Lee J., Lee Y.-H., Hun H.B. (2020). Effects of canine mesenchymal stem cells-derived exosomes in a mouse model of canine mammary tumor. J. Prev. Vet. Med..

[130] Luo T., Liu Q., Tan A., Duan L., Jia Y., Nong L., Tang J., Zhou W., Xie W., Lu Y. (2020). Mesenchymal Stem Cell-Secreted Exosome Promotes Chemoresistance in Breast Cancer via Enhancing miR-21-5p-Mediated *S100A6* Expression. Mol. Ther. Oncolytics.

[131] Zhao L., Gu C., Gan Y., Shao L., Chen H., Zhu H. (2020). Exosome-mediated siRNA delivery to suppress postoperative breast cancer metastasis. J. Control. Release.

[132] Rezaeian A., Khatami F., Keshel S.H., Akbari M.R., Mirzaei A., Gholami K., Farsani R.M., Aghamir S.M.K. (2022). The effect of mesenchymal stem cells-derived exosomes on the prostate, bladder, and renal cancer cell lines. Sci. Rep..

[133] Jahangiri B., Khalaj-Kondori M., Asadollahi E., Dizaj L.P., Sadeghizadeh M. (2022). MSC-Derived exosomes suppress colorectal cancer cell proliferation and metastasis via miR-100/mTOR/miR-143 pathway. Int. J. Pharm..

[134] Ning S., Chen Y., Li S., Liu M., Liu H., Ye M., Wang C., Pan J., Wei W., Li J. (2023). Exosomal miR-99b-5p Secreted from Mesenchymal Stem Cells Can Retard the Progression of Colorectal Cancer by Targeting FGFR3. Stem Cell Rev. Rep..

[135] Ren J., Liu Y., Yao Y., Feng L., Zhao X., Li Z., Yang L. (2021). Intranasal delivery of MSC-derived exosomes attenuates allergic asthma via expanding IL-10 producing lung interstitial macrophages in mice. Int. Immunopharmacol..

[136] Dou R., Zhang X., Xu X., Wang P., Yan B. (2021). Mesenchymal stem cell exosomal tsRNA-21109 alleviate systemic lupus erythematosus by inhibiting macrophage M1 polarization. Mol. Immunol..

[137] Zhou W., Zhou Y., Chen X., Ning T., Chen H., Guo Q., Zhang Y., Liu P., Zhang Y., Li C. (2021). Pancreatic cancer-targeting exosomes for enhancing immunotherapy and reprogramming tumor microenvironment. Biomaterials.

[138] Liu C., Yang T.-H., Li H.-D., Li G.-Z., Liang J., Wang P. (2023). Exosomes from bone marrow mesenchymal stem cells are a potential treatment for ischemic stroke. Neural Regen. Res..

[139] Zheng H., Liang X., Han Q., Shao Z., Zhang Y., Shi L., Hong Y., Li W., Mai C., Mo Q. (2021). Hemin enhances the cardioprotective effects of mesenchymal stem cell-derived exosomes against infarction via amelioration of cardiomyocyte senescence. J. Nanobiotechnol..

[140] Cao J.Y., Wang B., Tang T.T., Wen Y., Li Z.L., Feng S.T., Wu M., Liu D., Yin D., Ma K.L. (2021). Exosomal miR-125b-5p deriving from mesenchymal stem cells promotes tubular repair by suppression of p53 in ischemic acute kidney injury. Theranostics.

[141] Ma T., Chen Y., Chen Y., Meng Q., Sun J., Shao L., Yu Y., Huang H., Hu Y., Yang Z. (2018). MicroRNA-132, Delivered by Mesenchymal Stem Cell-Derived Exosomes, Promote Angiogenesis in Myocardial Infarction. Stem Cells Int..

[142] Zhang Z., Ge L., Zhang S., Wang J., Jiang W., Xin Q., Luan Y. (2020). The protective effects of MSC-EXO against pulmonary hypertension through regulating Wnt5a/BMP signalling pathway. J. Cell. Mol. Med..

[143] Ge L.L., Jiang W., Zhang S.S., Wang J., Xin Q., Sun C., Li K.L., Qi T.G., Luan Y. (2021). Mesenchymal Stromal Cell-derived Exosomes Attenuate Experimental Pulmonary Arterial Hypertension. Curr. Pharm. Biotechnol..

[144] Liu Z., Wu C., Zou X., Shen W., Yang J., Zhang X., Hu X., Wang H., Liao Y., Jing T. (2020). Exosomes derived from mesenchymal stem cells inhibit neointimal hyperplasia by activating the Erk1/2 signalling pathway in rats. Stem Cell Res. Ther..

[145] Suchorska W.M., Lach M.S. (2016). The role of exosomes in tumor progression and metastasis (Review). Oncol. Rep..

[146] Frediani J.N., Fabbri M. (2016). Essential role of miRNAs in orchestrating the biology of the tumor microenvironment. Mol. Cancer.

[147] Vallabhaneni K.C., Penfornis P., Dhule S., Guillonneau F., Adams K.V., Mo Y.Y., Xu R., Liu Y., Watabe K., Vemuri M.C. (2015). Extracellular vesicles from bone marrow mesenchymal stem/stromal cells transport tumor regulatory microRNA, proteins, and metabolites. Oncotarget.

[148] Zhou X., Li T., Chen Y., Zhang N., Wang P., Liang Y., Long M., Liu H., Mao J., Liu Q. (2019). Mesenchymal stem cell-derived extracellular vesicles promote the in vitro proliferation and migration of breast cancer cells through the activation of the ERK pathway. Int. J. Oncol..

[149] Meng Q., Zhang B., Zhang Y., Wang S., Zhu X. (2021). Human bone marrow mesenchymal stem cell-derived extracellular vesicles impede the progression of cervical cancer via the miR-144-3p/CEP55 pathway. J. Cell. Mol. Med..

[150] Xu Y., Shen L., Li F., Yang J., Wan X., Ouyang M. (2019). microRNA-16-5p-containing exosomes derived from bone marrow-derived mesenchymal stem cells inhibit proliferation, migration, and invasion, while promoting apoptosis of colorectal cancer cells by downregulating ITGA2. J. Cell. Physiol..

[151] Roccaro A.M., Sacco A., Maiso P., Azab A.K., Tai Y.T., Reagan M., Azab F., Flores L.M., Campigotto F., Weller E. (2013). BM mesenchymal stromal cell-derived exosomes facilitate multiple myeloma progression. J. Clin. Investig..

[152] Abu N., Rus Bakarurraini N.A.A., Nasir S.N. (2021). Extracellular Vesicles and DAMPs in Cancer: A Mini-Review. Front. Immunol..

[153] Goncalves J.P., Deliwala V.J., Kolarich D., Souza-Fonseca-Guimaraes F., Wolfram J. (2022). The cancer cell-derived extracellular vesicle glycocode in immunoevasion. Trends Immunol..

[154] Vakhshiteh F., Atyabi F., Ostad S.N. (2019). Mesenchymal stem cell exosomes: A two-edged sword in cancer therapy. Int. J. Nanomed..

[155] Sun Y., Liu G., Zhang K., Cao Q., Liu T., Li J. (2021). Mesenchymal stem cells-derived exosomes for drug delivery. Stem Cell Res. Ther..

[156] Tan F., Li X., Wang Z., Li J., Shahzad K., Zheng J. (2024). Clinical applications of stem cell-derived exosomes. Signal Transduct. Target. Ther..

[157] McLaughlin C., Datta P., Singh Y.P., Lo A., Horchler S., Elcheva I.A., Ozbolat I.T., Ravnic D.J., Koduru S.V. (2022). Mesenchymal Stem Cell-Derived Extracellular Vesicles for Therapeutic Use and in Bioengineering Applications. Cells.

[158] Vymetalova L., Kucirkova T., Knopfova L., Pospisilova V., Kasko T., Lejdarova H., Makaturova E., Kuglik P., Oralova V., Matalova E. (2020). Large-Scale Automated Hollow-Fiber Bioreactor Expansion of Umbilical Cord-Derived Human Mesenchymal Stromal Cells for Neurological Disorders. Neurochem. Res..

[159] Zhang A., Wong J.K.U., Redzikultsava K., Baldry M., Alavi S.K., Wang Z., van Koten E., Weiss A., Bilek M., Yeo G.C. (2023). A cost-effective and enhanced mesenchymal stem cell expansion platform with internal plasma-activated biofunctional interfaces. Mater. Today. Bio.

[160] Fu Y., Li J., Li M., Xu J., Rong Z., Ren F., Wang Y., Sheng J., Chang Z. (2022). Umbilical Cord Mesenchymal Stem Cells Ameliorate Inflammation-Related Tumorigenesis via Modulating Macrophages. Stem Cells Int..

